# Multiscale Modeling of Aqueous Electric Double Layers

**DOI:** 10.1021/acs.chemrev.3c00307

**Published:** 2023-12-20

**Authors:** Maximilian Becker, Philip Loche, Majid Rezaei, Amanuel Wolde-Kidan, Yuki Uematsu, Roland R. Netz, Douwe Jan Bonthuis

**Affiliations:** † Fachbereich Physik, Freie Universität Berlin, 14195 Berlin, Germany; ‡ Laboratory of Computational Science and Modeling, IMX, École Polytechnique Fédérale de Lausanne, 1015 Lausanne, Switzerland; § Institute of Theoretical Chemistry, Ulm University, 89081 Ulm, Germany; ∥ Department of Physics and Information Technology, Kyushu Institute of Technology, 820-8502 Iizuka, Japan; ⊥ PRESTO, Japan Science and Technology Agency, 4-1-8 Honcho, Kawaguchi, Saitama 332-0012, Japan; ∇ Institute of Theoretical and Computational Physics, Graz University of Technology, 8010 Graz, Austria

## Abstract

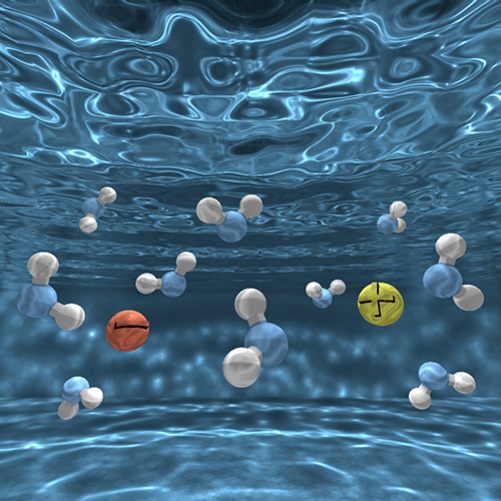

From the stability of colloidal suspensions to the charging of electrodes, electric double layers play a pivotal role in aqueous systems. The interactions between interfaces, water molecules, ions and other solutes making up the electrical double layer span length scales from Ångströms to micrometers and are notoriously complex. Therefore, explaining experimental observations in terms of the double layer’s molecular structure has been a long-standing challenge in physical chemistry, yet recent advances in simulations techniques and computational power have led to tremendous progress. In particular, the past decades have seen the development of a multiscale theoretical framework based on the combination of quantum density functional theory, force-field based simulations and continuum theory. In this Review, we discuss these theoretical developments and make quantitative comparisons to experimental results from, among other techniques, sum-frequency generation, atomic-force microscopy, and electrokinetics. Starting from the vapor/water interface, we treat a range of qualitatively different types of surfaces, varying from soft to solid, from hydrophilic to hydrophobic, and from charged to uncharged.

## 1 Introduction

In the context of charged objects in electrolyte solutions, the electric double layer is of special interest: it is the region where the charge on the objects’ surface is neutralized by oppositely charged species in the liquid solution. The charge on the surface can be caused either by electrochemical reactions, or by the preferential adsorption of charged species, or by the charging of a metallic interface. Despite its typically modest spatial extent, it is hard to overestimate the importance of the electrical double layer for the physical chemistry of aqueous solutions. In particular, every measurement of the electrostatic potential in solution is affected by the double layer at the electrode’s surface. Similarly, estimates of the surface charge density of solutes by means of their electrophoretic mobility are dominated by the double layer structure. Furthermore, all electrochemical reactions take place in the double layer, and, finally, the double layer mediates the interactions between charged solutes. This is of particular importance in biological and soft-matter systems, where electrostatics dominate the interactions between colloids, macromolecules and macromolecular assemblies, but also in battery technology, colloidal chemistry, micro- and nanofluidics, catalysis, and many other branches of science and industry.

**Figure 1:**
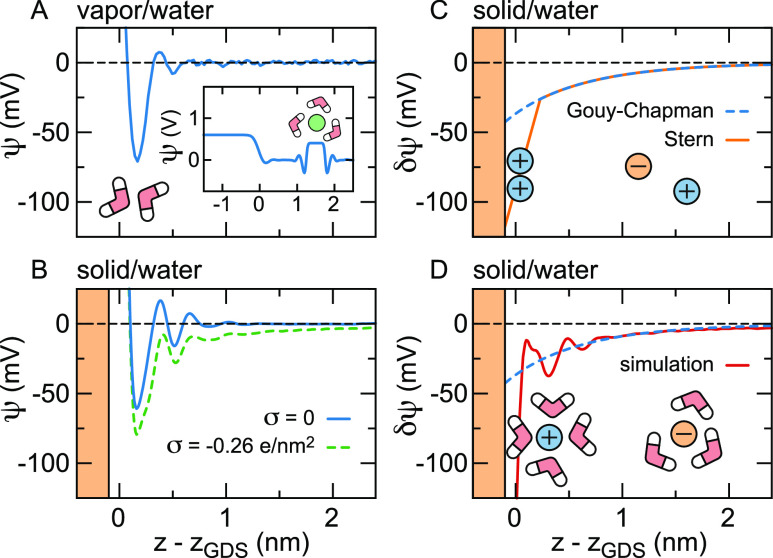
Electrostatic interface and double layer potentials from force-field (FF) molecular dynamics simulations and mean-field models. (A) Total potential ψ(*z*) at the vapor/water interface, reflecting the orientation of the water molecules, as well as higher-order multipole moments, see section 2. The inset shows the potential on a line through the interface and a solvated helium atom in the water bulk. (B) Potential at the silica–water interface at different surface charge densities σ. The shaded area indicates the surface up to the position of the outermost oxygen atoms at *z* – *z*_GDS_ = −0.1 nm, where *z*_GDS_ denotes the Gibbs dividing surface. (C) Potential difference δψ(*z*) between the charged and uncharged surface calculated using the Gouy–Chapman and Stern theories for σ = −0.26*e*/nm^2^. (D) δψ(*z*) at the silica surface extracted from the simulations shown in (B) together with the Gouy–Chapman theory. Models for δψ(*z*) are discussed in sections 3 and 4.

Apart from ions and surface charge, water molecules play a pivotal role in structuring the electric double layer. A clear illustration of the ordering and orientation of the interfacial water molecules is provided by the existence of a nonzero electrostatic potential ψ(*z*) also in the absence of surface charge, such as the potential observed in molecular dynamics simulations of the vapor/water interface, see Figure 1(A). The underlying interfacial structure is observed in experiments^1^ and a range of different simulations,^2,3^ but note that the electrostatic potential across an aqueous interface crucially depends on the molecular model used to simulate the water. A potential difference similar to the one observed at aqueous interfaces is found in the solvation shell of ions and neutral particles in aqueous solution, as shown in the inset of Figure 1(A).

The interfacial electrostatic potential is modified by the presence of ions and a surface charge density σ, as shown in Figure 1(B) for a silica surface in a 0.15 M NaCl solution. We refer to the difference between the potential with and without surface charge and ions as δψ(*z*). Throughout the 19th century, there has been a steady improvement of the understanding of ion distributions at charged surfaces, culminating in the formulation of the mean-field Poisson–Boltzmann equation for δψ(*z*) by Gouy^4^ and Chapman,^5^ which still forms the basis of most current double layer theory, see Figure 1(C). Since Gouy–Chapman, several modifications of the Poisson–Boltzmann theory have been proposed. First of all, the observation that the Gouy–Chapman model overestimates the interfacial capacitance of a Ag electrode in a AgNO_3_ solution resulted in the introduction of the Stern layer,^6^ see Figure 1(C). Although the interpretation of the Stern layer is still subject to debate,^7−9^ most models agree that it constitutes a combination of specific ion adsorption and a reduced dielectric constant as originally postulated by Stern. For instance, a Stern-layer like contribution to the capacitance at metal surfaces has been shown to arise from the nonlocal dielectric response function.^10^ Second, the finite size of ions has been introduced both to allow for ion-specificity and to prevent the unphysically high interfacial ion densities obtained using the Gouy–Chapman theory.^11−15^ Third, it has been realized that ion-specific effects not only depend on ion size, but there are additional contributions from the ion–surface and ion–water interactions. One way to include these effects into the Gouy–Chapman theory is by adding an ion-specific and ion-position dependent free energy to the electrostatic potential energy in the Boltzmann terms.^16−18^ Fourth, as mentioned above, the water structure around the ions and around the solutes is not homogeneous. Instead, water molecules around hydrophobic solutes,^19^ amino acids,^20^ lipid membranes^21^ and ions^22^ experience structural and dynamic constraints. Analytical theory predicts that the orientational constraints on water molecules at aqueous interfaces lead to a spatially varying dielectric response.^23^ Moreover, due to the correlations of the water polarization in time and space, the bulk dielectric response needs to be expressed as a function of wave vector^24^ and frequency,^25^ with consequences for the electrostatic screening around charged solutes^26−28^ and in confinement.^29,30^ At interfaces, including the effect of the boundary on the nonlocal spatial correlations results in an effective dielectric response that varies with the distance from the interface.^31^ As an alternative to including the dielectric through a response function, it can be taken into account by explicitly accounting for the presence of dipolar particles.^32−34^

Several approaches beyond these mean-field continuum theories are being actively pursued. Classical density functional theory relies on the minimization of the grand potential written as a functional of the one-particle density, which for rigid dipolar particles is a function of position and orientation.^35^ Free energy functionals have been designed to include microscopic effects such as the coupling between polarization and solvent densities, dipolar saturation and the nonlocal character of the dielectric response discussed above.^36,37^ The contribution to the free energy functional stemming from the interactions within the fluid depends on the fluid’s direct correlation function, which is connected to the pair correlation function through the Ornstein–Zernike equation.^35^ Integral equation theories provide a way to estimate these correlation functions by solving the Ornstein–Zernike equation together with a closure relation typically based on molecular pairwise additive interaction potentials. Integral equation theories can accurately predict the structure of liquid water, from which the nonlocal dielectric response function has been derived.^38^ Many other analytical approaches exist,^39^ yet formulating an analytical theory with sufficient molecular details that agrees with experiments—which is essential in order to explain experimental observations in terms of the molecular structure of the interface—has so far proved to be an insurmountable challenge.

The advent of molecular dynamics (MD) simulation techniques has brought a new perspective, providing insight into the interfacial structure at the atomic and subatomic scale. In particular, simulations can be used to study a number of phenomena not covered by the mean-field description. First, molecular dynamics simulations naturally include fluctuation and correlation effects between the particles, providing an important addition to mean-field models. One example of a phenomenon depending on these effects is like-charge attraction,^40−43^ which cannot be described within the mean-field approach. Second, the electrostatic structure of the water molecules can be modeled beyond ideal multipole moment approximations, which turns out to be of immense importance for the interfacial water structure. The detailed molecular electrostatic structure is incorporated in the simulations through the design of the pairwise interaction potential in force-field-based (FF) molecular dynamics simulations and through the approximation of the electron density in quantum density functional theory (DFT)-based molecular dynamics simulations. Third, simulations can be used to study the image charge interaction experienced by ions approaching a dielectric boundary, which is not included in Gouy–Chapman theory. Simulations have been used in particular to study how image charge effects depend on the dielectric properties of the water,^44,45^ the ions^46^ and the surface.^47^ Fourth, ion-specific effects on adsorption, interfacial structure, and solvation structure can be studied in greater detail. An important prerequisite in the case of FF-based MD simulations is that an accurate method for the parametrization of the ionic interaction potentials is available.^48^ Finally, DFT-based simulations of charged interfaces can be used to study the coupling between surface charges and chemical reactivity at metal oxide interfaces.^49−51^ The combined effects of these atomistic aspects on the electrostatic potential δψ(*z*) at a silica surface carrying a fixed surface charge density in contact with a 0.15 M NaCl solution, calculated using FF-based MD simulations, can be seen in Figure 1(D). Conspicuous differences between the simulated *δ*ψ(*z*) and the Gouy–Chapman prediction include the steep increase near the surface—which, incidentally, is also present in the heuristic Stern model,^6^ see Figure 1(C)—and the nonmonotonic behavior in the first nanometer. The purpose of the present Review is to discuss the origins and consequences of these features of ψ(*z*) and δψ(*z*) from the molecular scale up to the scale of colloids, nanofluidic devices and electrodes.

In this Review, we discuss the developments in the multiscale modeling of electric double layers over the last two decades, focusing on soft hydrophobic surfaces such as vapor/water and oil/water, solid hydrophilic surfaces such as silica, more hydrophobic solid surfaces such as graphene, and soft hydrophilic surfaces such as lipid membranes. We treat both uncharged and charged surfaces, expanding on a recent review of the latter.^52^ First, we discuss water density oscillations and molecular orientation, comparing FF- and quantum DFT-based MD simulations. Second, we describe the implications of this interfacial structure on the electrostatic properties of the electric double layer. In particular, we discuss the interface potential at vapor/water surfaces and the dielectric tensor at different types of interfaces. Third, as a separate, but closely related, topic, we discuss the effects of the interfacial structure on the local interfacial effective viscosity. Nanofluidic experiments reveal anomalous transport properties at interfaces and in confinement.^53^ The viscosity is affected by the same interfacial structure affecting the dielectric tensor,^54^ and studying both the electrostatic and hydrodynamic properties of electric double layers gives complementary information on this structure. The hydrodynamic effects are particularly relevant for electrokinetic measurements, which play a prominent role in investigations of the double layer properties.^55−57^ Most notably, electrokinetic measurements are used to estimate the ζ-potential. The ζ potential is often interpreted as a measure of the electrostatic surface charge and potential,^58^ which we show to be a problematic interpretation in some cases. Fourth, we discuss the effects of the interfacial structure on ions in the solution. In particular, we show how the dielectric properties, the image charge potential and the potential of mean force affect the ion density at uncharged and charged surfaces, with an emphasis on the different ways in which the results from molecular simulations can be incorporated in the Poisson–Boltzmann theory.^16,59^ Finally, we review how the presence of minute amounts of surfactants can affect experimental measurements at hydrophobic surfaces and their theoretical interpretation. Combining simulations and continuum theory allows the construction of a multiscale description of aqueous interfaces in general and electric double layers in particular, spanning length scales from the subnanometer details of the quantum DFT-based simulations to the micrometer scale that can be easily reached using numerical mean-field calculations.

## 2 Molecular Structure of the Pristine Water Interface

Without any surface charge or ions present, aqueous interfaces still exhibit a strong variation of the electrostatic potential due to the layering and orientation of the water molecules. Although pristine interfaces are rare in practice, the effect of the presence of an interface on the water structure is so strong that a theoretical consideration of pristine aqueous interfaces is warranted.

### 2.1 Density Variations

When packing hard spheres or Lennard-Jones particles next to a solid surface, density oscillations are typically expected, and the same is true for water at flat solid surfaces. Early measurements of the force between curved hydrophilic mica surfaces using a surface force apparatus show very strong oscillations,^60^ attributed to a layered water structure. Also at planar surfaces, atomic force microscopy (AFM) experiments reveal oscillatory force profiles at hydrophobic graphite and hydrophilic mica surfaces using a silicon AFM tip,^61^ as well as at various hydrophobic surfaces, including graphite, using a hydrophobic carbon AFM tip.^62^ Water-mediated hydration forces are caused not only by molecular density fluctuations. The coupling between polarization and molecular density fluctuations gives rise to a resonance peak in the wave-vector-dependent nonlocal dielectric response function, which in turn causes an oscillatory decay in the water-mediated forces between smooth surfaces.^63^ Yet to show that the oscillatory structure is present also in the absence of the AFM tip, the interfacial density of water oxygen can be extracted from a fit to X-ray spectroscopy data. At mica surfaces, X-ray measurements show density oscillations with the first peaks reaching more than twice the bulk density.^64^ Although the layering is typically smeared out at soft and rough surfaces, the interfacial density can still be different from the bulk, varying from depletion at hydrophobic surfaces^65^ to enhancement at hydrophilic ones.^66^

The density variations affect the electric double layer in multiple ways. First, the interfacial effective viscosity and surface friction are directly related to the interfacial density. Second, an enhanced or reduced density of polar molecules affects the dielectric properties of the interfacial layer. Third, density variations have a direct effect on the adsorption of ions due to steric exclusion.^67^ In particular, FF MD simulations show that ions at charged hydrophobic surfaces accumulate between the peaks in the oscillating water density profile,^68^ an effect expected to vanish for surfaces exhibiting a roughness of as little as the size of a molecule.^63^ In these simulated density profiles, the traditional interfacial layers can be clearly identified,^7^ viz., partially dehydrated ions between the surface and the first density peak form the inner Helmholtz layer of adsorbed ions, fully hydrated ions between the first two density peaks form the outer Helmholtz layer, and the rest of the fluid forms the diffuse layer. These aspects will be discussed in more detail in section 3.

### 2.2 Orientation

Apart from forming a layered structure, interfacial water molecules adopt a specific orientation because of their strong polarity. One of the simplest water models exhibiting polar ordering is a Stockmayer fluid, consisting of Lennard-Jones spheres carrying point dipoles. Because of its symmetry, a particular orientation perpendicular to the uncharged interface is not expected. Instead, molecules at the vapor/liquid interface of a Stockmayer fluid orient parallel to the interface.^69,70^ In contrast, including higher-order multipole moments in the description of the water molecules does allow for a symmetry breaking perpendicular to the interface. For example, modeling water molecules as a point dipole and point quadrupole inside a spherical dielectric cavity leads to an interfacial polarization with hydrogen atoms pointing toward the liquid phase.^71^ As the authors note, however, this prediction crucially depends on the assumed position of the multipoles in the spherical dielectric cavity, and the sign of the interfacial orientation can be reversed by shifting the multipoles with respect to the cavity. The bulk properties of water have been reproduced successfully by including multipole moments up to the octupole,^72^ but the effects of multipole moments above the quadrupole on the interfacial orientation have not been explicitly investigated so far.

**Figure 2:**
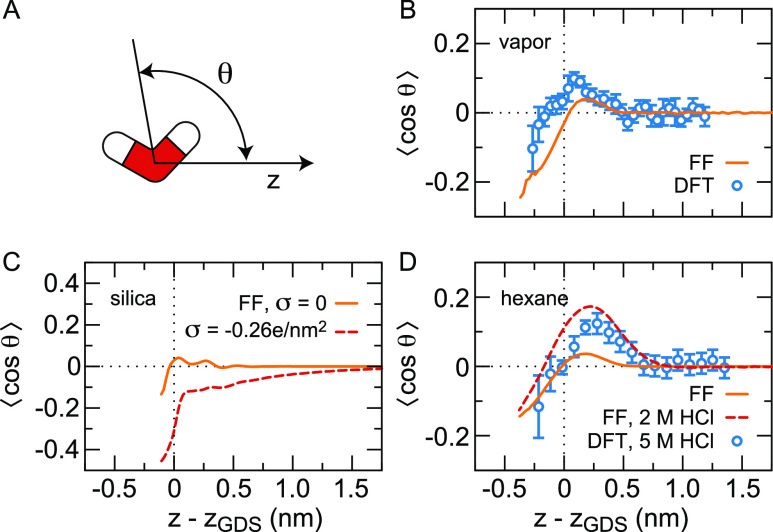
Water orientation at the vapor, silica, and hexane–water interface. (A) The angle θ is defined as the angle between the water dipole moment and the *z*-axis, which is perpendicular to the interface. The liquid aqueous phase is to the right of the Gibbs dividing surface. (B) ⟨cos θ⟩, where ⟨···⟩ denotes the lateral and time average, at the vapor/water interface from FF MD simulations (orange line) and DFT MD simulations (symbols). Data are shown for water densities above 0.1% of the bulk density. The DFT standard deviations have been obtained from block averaging in the time domain.^73^ (C) ⟨cos θ⟩ at the silica–water interface from FF MD simulations at surface charge density σ = 0 and at σ = −0.26*e*/nm^2^ and 0.15 M NaCl.^74,75^ (D) ⟨cos θ⟩ at the hexane/water interface from DFT-based MD simulations in the presence of 5 M HCl (symbols), as well as from FF MD simulations at both the pristine hexane/water interface (orange line)^76^ and in the presence of 2 M HCl, modeled as H_3_O^+^ and Cl^–^ ions (red broken line).^77^ The orientation of H_3_O^+^ is not included in ⟨cos θ⟩.

In the simplest FF-based MD simulations, water molecules are modeled as two positively charged hydrogen atoms rigidly attached to a negatively charged oxygen atom. Lennard-Jones interactions typically only act between the oxygen atoms, such as in SPC/E^78^ and TIP3P.^79^ In Figure 2, we compare the water orientation obtained from FF-based MD simulations using the SPC/E water model with the results from DFT MD using the BLYP density functional with Grimme dispersion corrections, GTH pseudopotentials, and either a DZVP-SR-MOLOPT (panel B)^73^ or a TZV2P (panel D)^77^ basis set. The orientation of the water molecules at the interface can be quantified by the angles of the OH vectors or the dipole vector with the surface normal.^80^ Here, we focus on the average cosine of the angle θ between the water dipole and the surface normal, see Figure 2(A). In Figure 2(B), we show the profile of ⟨cos θ⟩ as a function of the distance *z* to the Gibbs dividing surface (*z*_GDS_) at the vapor/water interface. On the vapor side of the Gibbs dividing surface, *z* – *z*_GDS_ < 0, we find ⟨cos θ⟩ < 0, indicating a molecular orientation with the hydrogen atoms pointing toward the vapor phase. From the Gibbs dividing surface, a layer of water molecules that are predominantly oriented with the hydrogen atoms pointing away from the vapor extends about 0.5 nm into the liquid water phase. Beyond this layer, the orientation becomes isotropic. FF and DFT simulation methods give qualitatively similar results, yet the orientation profile found in DFT-based simulations is slightly more pronounced. Previous calculations of the water orientation at the vapor/water interface based on a separate analysis of the two OH angles in DFT-based simulations are in excellent agreement with the results shown here.^3,81^ Although otherwise similar, however, the orientation of water dipoles with hydrogen atoms pointing toward the vapor has not been observed in FF-based simulations using TIP4P water.^82^ Using a classical density functional theory treating dipolar molecules as spherical shells with a dipolar charge distribution, simulated interfacial orientations of a number of different polar fluids confined between charged surfaces have been reproduced, confirming the interplay between the molecule’s intramolecular charge distribution, its finite size and its orientation.^83^

In experiments, the water orientation can be measured using sum-frequency generation (SFG), because only groups of molecules with a net polar orientation contribute to the sum-frequency spectrum.^84^ The sign of the imaginary part of the second-order susceptibility χ^(2)^, which can be detected in phase-sensitive SFG experiments, is determined by the direction of the net polar orientation.^85^ Analysis of the vapor/water interface using phase sensitive sum-frequency spectroscopy shows one sharp positive peak around 3700 cm^–1^ and a negative region at 3200–3600 cm^–1^.^86,87^ The positive peak has been interpreted as coming from water molecules in the outermost water layer, having one OH group which is not hydrogen-bonded pointing toward the vapor phase, and the negative region as coming from the donor-bonded OH groups pointing into the fluid phase.^88^ A very similar SFG signal is found at the weakly interacting graphene/water interface.^89^ This experimentally determined water orientation evidently agrees very well with the orientation profile determined by the FF- and DFT-based MD simulations shown in Figure 2(B), yet in the absence of polarizability a quantitative comparison with FF simulations is out of reach. A direct comparison of the SFG spectrum with FF simulations can be made when polarizable water molecules are used instead.^90^ At the vapor/water interface, the comparison is qualitatively good,^91^ but also for polarizable water models a quantitative agreement is not obtained. Good agreement with experimental SFG spectra can be obtained from DFT-based MD simulations, however, depending on the level of theory used.^92^

Silica simulations have been performed using the force field developed by Emami et al.,^93^ using the Q3 form of silica, which has 4.7 silanol groups per nm^2^.^75^ The deprotonation levels are set at 0 and at 5%, the latter corresponding to approximately pH 5, having a surface charge density of −0.26*e* nm^–2^. For the electrolyte, the SPC/E water model^78^ and the ion force field from Loche et al.^48^ have been employed. At the silica interface, the orientation profile shows the same qualitative features as the one at the vapor/water interface, but the region of negative ⟨cos θ⟩ is less extended because of the presence of the solid surface and the region of positive ⟨cos θ⟩ exhibits oscillations reminiscent of those in the density profile, see Figure 2(C). Upon adding a negative surface charge, together with counterions, in addition to a moderate background concentration of 0.15 M NaCl, ⟨cos θ⟩ shifts down and becomes negative in the entire interface region. That means that the water orientation with hydrogen atoms pointing toward the surface becomes more pronounced, as expected based on the electric field emanating from the charged surface. The orientation profile at the pristine hexane interface very closely resembles the orientation profile at the vapor/water interface, see Figure 2(D). Similar to the situation for TIP4P at the vapor/water interface, orientation of the water dipole with hydrogen atoms toward the alkane is not observed in simulations of the pristine octane/water interface using TIP4P/2005.^94^ Sum-frequency spectroscopy reveals a strong orientation with the OH groups pointing toward the surface at quartz/water interfaces,^95^ silica/water interfaces^96^ and zwitterionic lipid membranes,^97,98^ as well as alkane/water and PDMS/water interfaces,^99^ yet the contribution from the reverse orientation observed at the vapor/water interface, as well as in the simulations at silica and hexane, is typically absent. The reason for these inconsistencies is unclear. Apart from the simulations at silica and hexane shown in Figure 2(C–D), the orientation of the outermost water layer with the hydrogen atoms toward the surface is reproduced in FF^100^ and DFT^101^ simulations of phospholipids.

The presence of ions in the interfacial region can have a strong effect on the orientation profile. One ion type exhibiting adsorption to the vapor/water interface is the hydronium ion, which itself adopts a pronounced orientation at the interface.^77^ Adding 2 M of HCl to the hexane–water interface gives rise to a significantly enhanced water orientation in the region of positive ⟨cos θ⟩, see Figure 2(D). This enhanced profile agrees well with DFT-based simulations at an even higher HCl concentration of 5 M. At the silica surface, SFG spectra exhibit a strong dependence on the concentration and the nature of the cations present in the electrolyte.^102^ Whereas this dependence has previously been attributed to variations in the surface potential,^103^ a recent combination of SFG spectroscopy and MD simulations shows that the presence of cations leads to an ion-specific change of orientation of the water molecules in the first nanometer adjacent to the surface instead.^74^ Other changes to the composition of the interfacial region also modify the interfacial orientation. For example, FF MD simulations indicate that the presence of charged surfactants affects the water orientation profile at air/water and oil/water surfaces.^76,104^ Although molecules in the outer water layer maintain their orientation with the dipoles away from the aqueous phase, the orientation in the subsurface layer is sensitive to the presence of the surfactants, with the negatively charged SDS and the positively charged C_12_TAB having opposite effects.^76^ A qualitatively similar reversal of the water orientation has been observed in SFG spectra.^98,105^ At zwitterionic lipid membranes, the water orientation can also be reversed by inverting the polarity of the headgroup, as revealed by SFG spectroscopy and FF MD simulations at DOPC and DOCPe lipids.^100^

### 2.3 Interface Potential

Directly related to the interfacial orientation, an electrostatic potential drop arises across the aqueous interface. There are, however, at least two different ways in which such a potential drop can be defined. First, the potential drop can be defined as the volume-averaged electrostatic potential in the bulk water with respect to the vacuum. This potential drop, referred to as the mean inner electrostatic potential, can be evaluated experimentally using electron diffraction^106^ or electron holography.^107^ In these measurements, the response of the electrons in the aqueous solution to the beam electron can be neglected because of the high energy of the latter.^106^ Second, the surface potential drop can be defined electrochemically as the potential experienced by a charged particle, such as a hydrogen or chloride ion, upon crossing the interface. In contrast to the mean electrostatic potential, the electrochemical potential includes the effect of a possibly significant perturbation of the structure of the solution due to the charged particle.

**Figure 3:**
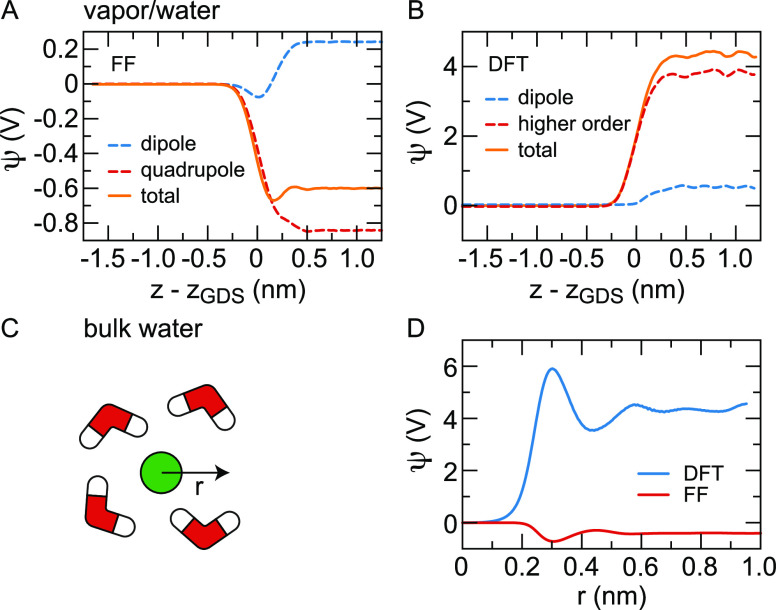
Electrostatic potential at the vapor/water interface. (A) Surface potential from force-field-based molecular dynamics simulations split into dipolar and quadrupolar terms.^73^ (B) Surface potential from molecular dynamics simulations based on quantum density functional theory split into dipolar and higher-order terms according to eqs 1, 2 and 3.^73^ (C) Schematic drawing of a cavity (green) solvated in bulk water. (D) Cavity potential as a function of the radial distance *r* from the center of a cavity formed by a helium atom using FF- and quantum DFT-based molecular dynamics simulations.^73^

In both cases, the electrostatic potential ψ(***r***) across the interface can be calculated from simulations by integrating the electric field ***E***(***r***) between two positions ***r***_0_ and ***r*** located on opposite sides of the interface,
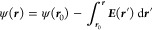
1For a more detailed insight, we split the electric field in the displacement field ***D***(***r***) and the polarization density ***m***(***r***),
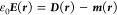
2The polarization can be expressed as a multipole expansion
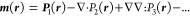
3with ***P***_1_(***r***) being the dipole density, *P*_2_(***r***) being the quadrupole density, etc. By inserting eqs 2 and 3 into eq 1, we find that at a planar interface in the absence of a monopole density, only two terms contribute to the potential drop across the interface: the dipole and the quadrupole. The dipolar term is directly related to the interfacial orientation. In Figure 3(A–B), the results of FF- and DFT-based simulations of the vapor/water interface show that the dipole contribution is positive, which is associated with molecules pointing their hydrogen atoms toward the aqueous phase.^73,108^ Despite the outer layer being oriented in the opposite direction, pointing with the hydrogen atoms toward the vapor, the molecules oriented with their hydrogens toward the water phase dominate the dipolar contribution to the potential because the water molecules in the subsurface layer are more numerous. The value of the dipole contribution is 0.24 V in the FF simulations. In DFT-based MD simulations, the dipole contribution is calculated from the positions of the nuclei and the Wannier centers of the electron distribution. The resulting value of 0.54 V is considerably higher than the value found in the FF simulations,^73^ which is directly related to the more pronounced orientation profile shown in Figure 2(B). The quadrupole density *P*_2_(***r***) is related to the intramolecular charge distribution and the density of water molecules and is also nonzero in bulk water. In FF-based MD simulations, the quadrupole density is negative and larger than the dipole contribution at the vapor/water interface, see Figure 3(A), and at diamond/water surfaces.^108^ The contribution from higher-order terms in the DFT-based simulations, obtained by subtracting the dipole contribution from the total potential, is also significantly larger than the dipole contribution but positive, see Figure 3(B). We will discuss this stark difference between DFT- and FF-based MD simulations below. Apart from dominating the total potential difference, the quadrupole contribution is of primary importance for the interfacial structure. For pure dipoles, for example, there is no energetic difference between a molecular orientation with the hydrogens pointing toward or away from the water phase at the vapor/water interface because the squared electric field around the dipole is independent of the dipole orientation. Adding the quadrupole term decreases the squared field strength on one side of the molecule while increasing it on the other side, which in combination with a dielectric discontinuity leads to a preferential orientation.

To calculate the mean inner potential difference between the vapor and the water bulk, ψ(***r***) and ψ(***r***_0_) are averaged over planes parallel to the interface located at *z*_*v*_ and *z*_*w*_ in the vapor and water bulk phases, respectively. Importantly, electron scattering also probes the inner-atomic regions of the water molecules, so the high positive and negative charge densities inside the atoms need to be included in the average. Therefore, calculating the potential difference from all-electron quantum density functional theory is expected to produce an accurate estimate of the mean inner potential difference. The total electrostatic potential extracted from our DFT MD simulations of the vapor/water interface gives a value of ψ(*z*_*w*_) – ψ(*z*_*v*_) = 4.35 ± 0.06 V, see Figure 3(B).^73^ Other literature values range from 3.1 to 4.3 V.^109−111^ Recent measurements of the mean inner potential based on electron holography give 3.5 V in a water droplet^112^ and 4.48 V at the vapor/water interface,^113^ which is indeed close to the values extracted from DFT MD simulations.

To calculate the electrochemical potential difference, the point ***r*** in eq 1 is located inside a particle. Important differences with the calculation of the mean inner potential are that the water’s inner-atomic regions are inaccessible to the particle and that the particle creates a second interface when immersed in the bulk water. As water molecules exhibit an anisotropic orientation also around an uncharged particle, this second interface gives rise to an additional potential difference.^38,114^ The potential difference between the vacuum and the interior of a particle in the bulk can therefore be split into a potential drop across the unperturbed vapor/water interface and a potential drop across the solvation shell of the particle, see Figure 1(A). When adding the two terms, the contributions from the inner-atomic regions cancel out, so also FF MD simulations are expected to be able to produce a good estimate of the electrochemical potential difference. For the first of the two terms, the potential drop across the unperturbed vapor/water interface, FF MD simulations yield ψ(*z*_*w*_) – ψ(*z*_*v*_) = −0.60 ± 0.002 V for SPC/E water,^73^ equal to the value obtained in other recent simulations.^114^ Other water force fields give rather different values: −0.13 V for TIP4P^115^ and −0.52 V for TIP3P.^116^ For comparison, the potential difference at the vapor/water interface has been calculated from DFT simulations, averaging over a volume in the bulk fluid excluding regions of high electron density.^110^ Depending on the exclusion cutoff, this procedure reduces the calculated potential difference from large positive values to −0.3 V, which is in the same range as the values from the FF MD simulations. This calculation confirms that the difference between the FF- and DFT-based interfacial potential drop originates in the higher-order multipole moments of the inner-atomic regions and is irrelevant for ion distributions and electrochemical applications.

For the second part of the electrochemical potential difference, the potential drop across the solvation shell of a particle, the potential is calculated as a function of the radial coordinate *r* with the origin inside a cavity formed by a helium atom solvated in water, see Figure 3(C). Figure 3(D) shows the resulting potential ψ(*r*) calculated using FF- and DFT-based MD simulations. The DFT MD yields a potential drop of ψ(*r*_*w*_) – ψ(*r*_*c*_) = 4.56 ± 0.08 V, with *r*_*c*_ = 0 denoting the position at the center of the cavity and *r*_*w*_ denoting a position in the water at a large radial distance from *r*_*c*_. In the FF MD, the potential drop across the solvation shell of the helium atom equals ψ(*r*_*w*_) – ψ(*r*_*c*_) = −0.40 ± 0.003 V, which is similar to the values of −0.3575 to −0.4057 V obtained for cavities of the size ranging from sodium to iodide ions.^114^ Adding the contributions from the vapor/water and the cavity/water interface and using ψ(*r*_*w*_) = ψ(*z*_*w*_), we find for the electrochemical potential difference between the center of the cavity and the vacuum ψ(*r*_*c*_) – ψ(*z*_*v*_) = −0.20 ± 0.004 V for the FF-based MD simulations and ψ(*r*_*c*_) – ψ(*z*_*v*_) = −0.2 ± 0.1 V for the DFT-based simulations.^73^ The good agreement between these values indicates that the two methods produce similar electrostatic structures, despite the 10% higher density of water in equilibrium with its vapor in the DFT simulations compared with the FF-based simulations and the difference in dipolar orientation at the interface shown in Figure 2(B).

**Figure 4:**
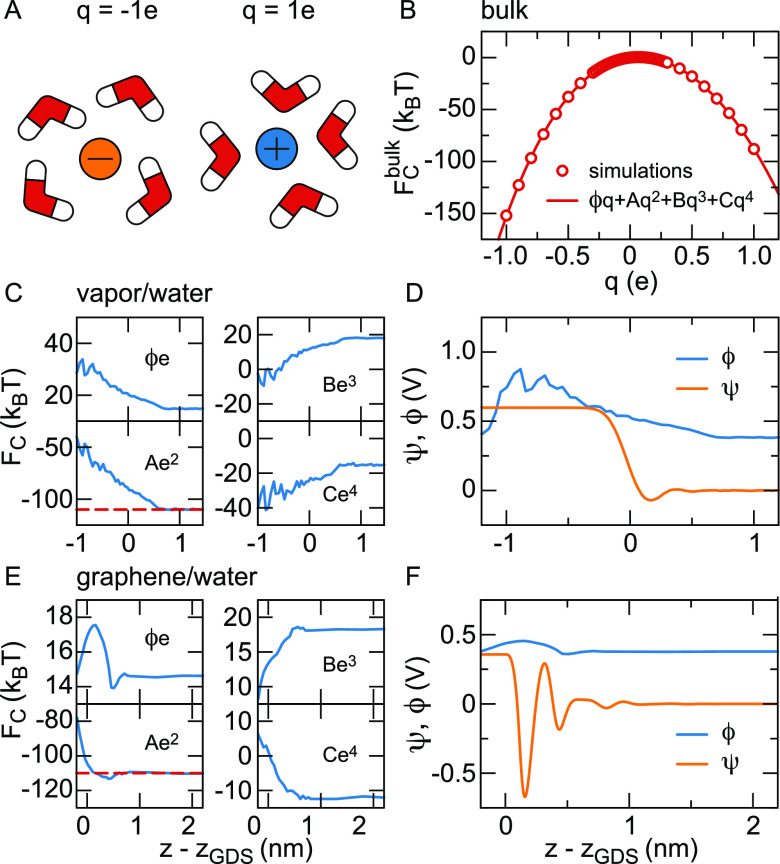
Solvation free energy from FF MD simulations. (A) Sketch of finite-sized particles with charge *q* in bulk water. The particle corresponds to Cl^–^ when *q* = −1*e*, and apart from the charge the particle does not change when *q* is increased to +1*e*. (B) Electrostatic energy of the particle in bulk water as a function of its charge *q* fitted with eq 4.^117^ (C–F) Terms of the polynomial fit according to eq 4 as a function of the distance to the Gibbs dividing surface *z*_GDS_ at (C, D) the vapor/water interface^118^ and (E, F) the graphene/water interface.^117^ The potential drop across the solvation shell is denoted by ϕ(*z*), *A*(*z*) is the prefactor of the Born solvation and *B*(*z*) and *C*(*z*) are nonlinear terms. The bulk value *A*(*z*_*w*_) is shown as a broken red line. In panels (D) and (F), we show the interface potentials ψ(*z*) without particle^119^ (also shown in Figure 3(A) for the vapor/water interface), together with the fitted potentials ϕ(*z*) inside the particle. For the boundary condition on the right-hand side of the figure, the electrostatic potential ψ(*z*_*w*_) in the unperturbed bulk water has been set to zero, which means that ϕ(*z*_*w*_) = 0.4 V equals the bulk cavity potential.

As an alternative approach to calculating the potential drop across the solvation shell, the potential felt by an ion can be calculated based on free energy calculations. For an ion of the size of chloride in bulk water, the electrostatic part of the solvation free energy is calculated from a thermodynamic integration in FF MD simulations.^117^ Schematic representations of a cation and an anion solvated in water are shown in Figure 4(A). In Figure 4(B) we show the Coulombic part of the free energy of an ion in bulk water, *F*_C_^*bulk*^, as a function of the ionic charge *q*. Instead of being a purely quadratic function of *q* as predicted by the Born solvation theory, the curve is asymmetric around *q* = 0, indicating a favorable solvation of anions.^120−122^ The asymmetric free energy for monovalent cations and anions is reproduced using the dielectric response from the atomic correlation functions, where the atomic correlation functions are obtained from integral equation theory.^38^ To model the asymmetry, the electrostatic part of the free energy is expanded in terms of the ionic charge *q* as
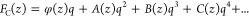
4The first term comes from the electrostatic potential drop across the solvation shell, and the second term comes from the Born solvation free energy. The higher-order terms in the expansion are caused by the nonlinear effect of the charge on the molecular structure of the solvation shell, with the uneven terms reflecting the systematic differences in the solvation of cations and anions. More specifically, the nonlinearity is caused by reorientation of the water molecules, which is an uneven function of the ion charge, and translational distortion of the solvent structure (electrostriction), which is an even function of the ion charge. Electronic polarization, which is absent in the simulations, is not considered here. Fitting the curve in Figure 4(B) with the expression of eq 4 yields the parameters ϕ, *A*, *B* and *C* in bulk, where the dependence on *z* has been dropped. The fit gives a potential inside the cavity of ϕ = 0.4 V with respect to the averaged potential outside the cavity, in agreement with ψ(*r*_*c*_) – ψ(*r*_*w*_) = 0.4 V calculated above.

To estimate the electrochemical potential difference felt by an ion as a function of the distance to the interface, the free energy *F*_C_(*z*) is calculated while restraining a chloride ion at a fixed position *z* relative to the interface. The fit values obtained by fitting eq 4 are shown in Figure 4(C) at the vapor/water interface and in Figure 4(E) at the graphene/water interface, multiplied by powers of the unit charge *e* to be able to plot all prefactors on the same scale. The potential drop across the solvation shell equals ϕ(*z*_*w*_)*e* = 15*k*_B_*T* at the bulk water position *z*_*w*_ in both cases, corresponding to ϕ(*z*_*w*_) = 0.4 V when the cavity is in bulk water, but the behavior near the interface depends strongly on the interface type, reflecting differences in the interfacial structure, spatial extent and rigidity. The Born solvation term equals *A*(*z*_*w*_)*e*^2^ = −110*k*_B_*T* in bulk and rises strongly in the interfacial region, reflecting the image charge repulsion that we will discuss in section 4.1. The observed variation of the nonlinear terms *B*(*z*) and *C*(*z*) when the ion approaches the interface, see Figures 4(C) and (E), can only be caused by the interaction between the induced field around the ion and the interfacial water structure. Since *B*(*z*) is the prefactor of an uneven function of *q*, the variation of *B*(*z*) accounts for the interaction between the water orientation around the ion and the interfacial ordering.^119^ Through the same logic, the change of *C*(*z*) is due to the interaction between the translational distortion of the hydration shell and the interfacial ordering. As can be seen in Figures 4(C) and (E), the variation of *C*(*z*) is qualitatively different indeed for solid and soft interfaces.^119^ The potential ϕ(*z*) felt by the ion, shown in Figures 4(D) and (F), shows a dramatic difference to the interface potential in the absence of the cavity, ψ(*z*). In particular, the strong variations of ψ(*z*) in the interfacial region are absent in ϕ(*z*). Regarding the potential shown in Figure 4(D), note that ions evaporating from the water to the vapor, or to other liquid phases, typically drag a part of their solvation shell with them,^123−125^ leading to large variations of the calculated ϕ(*z*) at *z* – *z*_GDS_ < −1 nm.^119,126^

In summary, considering the potential drop across the solvation shell of an ion shows that the electrochemical potential drop across the interface, ϕ(*z*_*w*_) – ϕ(*z*_*v*_), is significantly smaller than the mean inner potential difference as measured by electron diffraction and holography or calculated from DFT MD simulations. The electrochemical potential is also smaller than the potential drop calculated from FF MD simulations without cavity contribution. In contrast to linear electrostatic theory, the electrostatic free energy *F*_c_(*z*) comprises the cavity potential term ϕ(*z*)*q*, the Born term *A*(*z*)*q*^2^ and nonlinear contributions, all depending sensitively on the interface type.

## 3 Continuum Description of Interfacial Water

The molecular structure of the interfacial water layer discussed in the previous sections has a decisive influence on the electric double layer, and therefore on the thermodynamics, electrokinetics and hydrodynamics of macroscopic objects in solution. Yet many of these properties, such as the electrophoretic mobility of macroscopic colloids, the disjoining pressure between objects in solution, but also the surface tension at very low ion or surfactant concentration, are prohibitively expensive to simulate using molecular simulations. Therefore, a continuum description is paramount to use the results of the molecular dynamics simulations at macroscopic time and length scales. Using the Poisson equation for the electrostatics and the Stokes equation for the hydrodynamics, incorporating the molecular structure into the continuum description comes down to determining the dielectric and viscous response functions, as well as the nonelectrostatic interaction between ions and surfaces.

### 3.1 Dielectric Profile

Considering the restrictions imposed by a solid interface on the hydrogen bond network, a sharp decrease of the interfacial dielectric response has been predicted analytically.^23^ A local dielectric profile has been defined based on the nonlocal linear response tensor linking small increments of the electric and displacement fields.^31,127^ In molecular dynamics simulations, the local dielectric response tensor can be calculated from the response to a finite electric field^127,128^ or from the fluctuations of the polarization, which we will focus on here. Using a simulation volume *V* containing an interface with a normal vector in *z* direction, the inverse local dielectric profile perpendicular to the interface is calculated from^129^
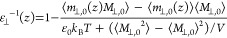
5with *m*_⊥,0_(*z*) and *M*_⊥,0_ being the polarization density at position *z* and the total polarization, respectively, perpendicular to the interface in the absence of an applied field. The second term in the denominator is caused by the periodic boundary conditions in *z* direction.^130^ Specifically, if a displacement field arises in *z* direction, the ensuing polarization *M*_⊥_ of the periodic images modifies the electric field. If the electrostatic periodic boundary conditions are turned off in *z* direction, using two-dimensional Ewald summation, for example, the second term in the denominator vanishes.^127^

**Figure 5:**
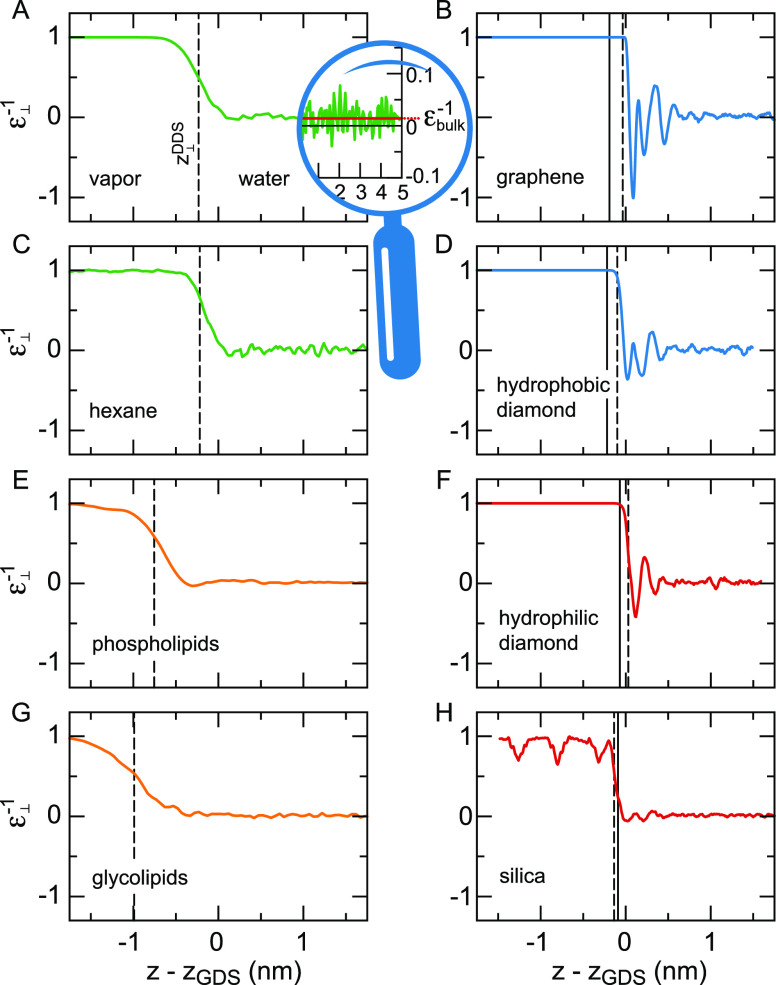
Perpendicular dielectric profiles at different solid and soft interfaces as a function of the distance with respect to the Gibbs dividing surface *z*_GDS_. Liquid water is on the right-hand side in each panel. The water dielectric profile for SPC/E converges to ε_⊥_^–1^(*z*_*w*_) = ε_*bulk*_^–1^ = 1/70, calculated as the average of the curve shown in the magnification in (A). Hydrophobic surfaces are shown in panels (A–D), and hydrophilic surfaces are shown in panels (E–H). Black solid lines indicate the position of the oxygen atoms of the surface OH groups at the hydrophilic solids and the position of the outermost carbon atoms at the hydrophobic solids. The dielectric dividing surface *z*_⊥_^DDS^ is shown as a broken black line. All partial charges in the fluid and the substrate are included in the calculation of the dielectric response. Data for each panel are from the indicated reference: (A),^126^ (B, G),^131^ (C),^118^ (D, F),^108^ (E)^132^ and (H).^75^ Except for panel (E), where TIP3P has been used, the curves have been obtained from simulations using the SPC/E water model.

Using eq 5, the predicted decrease of the interfacial dielectric response has been reproduced in FF MD simulations of pure water at uncharged surfaces.^108,127^ We show the inverse dielectric profiles of different solid and soft surfaces in Figure 5. For all surfaces, except possibly the glycolipids and the air–water interface, ε_⊥_^–1^(*z*) reverses sign several times. That means that the excess electric field vanishes where ε_⊥_^–1^(*z*) reaches zero and has the reverse sign in the regions where ε_⊥_^–1^(*z*) < 0, giving rise to several local minima in the electrostatic potential resulting from an applied external field. Regions of negative dielectric response, referred to as overscreening, are also found in the nonlocal dielectric function of bulk water.^26^ The effects of overscreening have also been observed in FF MD simulations of monovalent salt solutions at moderately charged silica surfaces^133^ and in classical density functional theory.^37^ The oscillatory features are very clearly visible at solid surfaces, whereas the dielectric profiles are smeared out at soft surfaces. The difference is partially caused by the fact that the dielectric profiles are convoluted with the interfacial density profiles due to capillary fluctuations. We attribute the pronounced oscillations at the graphene surface to the fact that its interface is atomically flat, in contrast to the other solid surfaces. In particular, the hydrophilic diamond surface features protruding hydroxide groups, and the silica surface consists of alternating silicon and oxygen atoms as well as hydroxide groups. Classical density functional theory has shown that smearing over the size of about 0.25 nm is already sufficient to suppress the oscillations in the ion density and hydration force resulting from the nonlocal dielectric response.^63^

The parallel dielectric profile is calculated from^127,130,134^
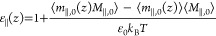
6with *m*_∥_(*z*) being the polarization density in the direction parallel to the interface. In contrast to the perpendicular dielectric profile, the profiles of ε_∥_(*z*) show that the parallel dielectric response is enhanced close to solid surfaces.^108,131^ The enhancement partially reflects the increased density of polar molecules, but also the effective water polarizability of the interfacial layer varies, depending on the surface type. In particular, whereas water at hydrophilic diamond surfaces exhibits a slight decrease of the parallel polarizability, the polarizability is enhanced at hydrophobic diamond surfaces^108^ and shows a giant increase inside carbon nanotubes.^135^

### 3.2 Box Profile of the Dielectric Constant

To simplify the description of the interfacial dielectric properties and to allow for analytical treatments,^136−139^ the dielectric profile can be modeled using a box profile. The box profile is designed to reproduce electrostatic potential differences across the interface on the macroscopic scale, whereas the microscopic dielectric details are lost in exchange for simplicity,
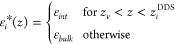
7with *i* being one of ⊥ or ∥ and *z*_*v*_ being a position in the nonaqueous phase. Either the interfacial dielectric constant ε_*int*_ or the dielectric dividing surface *z*_*i*_^DDS^ is a free parameter. Choosing, for example, ε_*int*_ = 1 unambiguously defines *z*_*i*_^DDS^ at the vapor/water interface, whereas setting *z*_*i*_^DDS^ at a solid surface defines the effective dielectric constant of the interfacial layer. The requirement for the construction of the box profile is that the potential difference between the surface and a point in the fluid far away from the interface is the same when using either the box profile or the full dielectric profile. That leads to the definition of the dielectric dividing surface
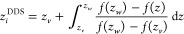
8with *z*_*v*_ and *z*_*w*_ being positions in the nonaqueous and aqueous phases, respectively. For the parallel dielectric dividing surface, we take *f*(*z*) = ε_∥_(*z*), while for the perpendicular dielectric dividing surface we take *f*(*z*) = ε_⊥_^–1^(*z*). Note that eq 8 is equivalent to the definition of the Gibbs dividing surface *z*_GDS_, where the water density is used for *f*(*z*), yet the position of the dielectric dividing surface typically does not coincide with the Gibbs dividing surface. The deviation between the two surface positions defines the interfacial dielectric excess and depends on the nature of the interface. In Figure 5, the dielectric dividing surface positions *z*_⊥_^DDS^ are shown as broken black lines. For the nonpolarizable solid surfaces, graphene and hydrophilic and hydrophobic diamond, *z*_⊥_^DDS^ is close to *z*_GDS_, yet there are small differences between the hydrophilic and the more hydrophobic surfaces. In particular, *z*_⊥_^DDS^ > *z*_GDS_ at the hydrophilic diamond surface, meaning that the interfacial water layer is less polarizable than expected based on the number density of water molecules, whereas the opposite situation is found at the hydrophobic diamond surface and the weakly interacting graphene. At the lipid interfaces and at silica, which are polarizable surfaces, the position of the dielectric dividing surface is determined to a large extent by the polarizability of the interfacial hydrophilic groups. Oil/water and vapor/water interfaces show almost identical dielectric behavior, as has been observed previously in simulations using united atoms.^140^

Using the dielectric profile based on the dielectric dividing surface of eq 7 as an alternative to using the complete dielectric profile, the interfacial capacitance of a large number of different surfaces can be accurately reproduced.^108,127^ For the scaling of the electrokinetic mobility with the bare surface charge density, the use of a layer of low dielectric constant as modeled by eq 7 is actually crucial to reproduce the experimental data.^137,141,142^ The two dividing surface positions for the response parallel and perpendicular to the surface are also important for the calculation of the image charge repulsion of ions at a dielectric boundary, as will be discussed in section 4.1.

### 3.3 Effective Dielectric Constant between Two Surfaces

To analytically calculate interactions between solutes and to compare to experiments, the dielectric profiles have to be replaced by effective dielectric constants of the medium where the measurement takes place. The effective dielectric constant between an uncharged atomic force microscope probe and a mica surface has been found to decrease over much larger distances than the roughly 1 nm suggested by simulations.^144^ Similarly, the effective dielectric constant of water films between cleaved mica platelets has been found to decrease over distances of the order of micrometers.^145^ More recently, the same effect has been measured between graphene surfaces.^143^

**Figure 6:**
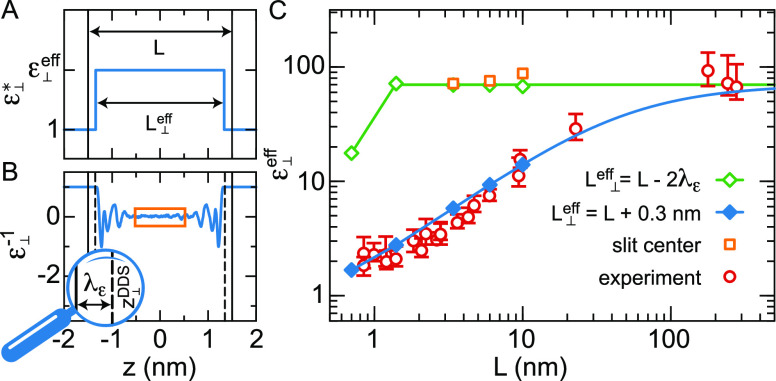
Effective perpendicular dielectric constant between two graphene sheets. (A) Approximate dielectric profile, eq 9. The distance between the graphene sheets is denoted by *L*. (B) Dielectric profile for *L* = 3 nm, with the saturated central part highlighted by the orange rectangle. The distance between the graphene sheet (solid black lines) and the dielectric dividing surface *z*_⊥_^DDS^ (broken black lines) is denoted by λ_ε_. (C) ε_⊥_^*eff*^ calculated using eq 12,^131^ compared to experiments.^143^ In the experiments, *L* is measured using atomic force microscopy. For the calculated curves, we use *L*_⊥_^*eff*^ = *L* – 2λ_ε_, which is the appropriate parametrization of eq 9 if ε_⊥_^*eff*^ = ε_*bulk*_, as well as *L*_⊥_^*eff*^ = *L* + 0.3 nm, which is determined by a fit to the experimental data. The diamond symbols are calculated from simulations in slits of width *L*,^131^ whereas the lines for *L* > 3 nm are calculated by inserting a bulk section with ε_⊥_^–1^ = 1/70 in the center of the simulated profile obtained at *L* = 3 nm. The orange squares show the dielectric constant in the center of the box calculated by averaging the dielectric profile over the central part of the box as indicated by the orange rectangle in panel (B).

To understand these results, we model the effective dielectric medium as a number of homogeneous dielectric materials in series, which for water between identical nonpolarizable surfaces becomes
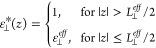
9see Figure 6(A). To calculate the effective dielectric constant ε_⊥_^*eff*^ of the medium, we require the electrostatic potential difference across the interfaces to be identical to the one calculated from the dielectric profile ε_⊥_^–1^(*z*). The relation between the perpendicular electric field increment δ*E*_⊥_(*z*) and the displacement field increment δ*D*_⊥_ reads
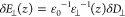
10with the electric field increment given by δ*E*_⊥_(*z*) = −∇_*z*_δψ(*z*). Integrating from −*L*/2 to *L*/2 using the dielectric profile ε_⊥_^–1^(*z*) and using the effective profile of eq 9 yields
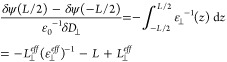
11Solving for ε_⊥_^*eff*^ gives
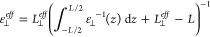
12Now there are different questions we may ask. The first one is what is the dielectric constant in the interior of the water slab depending on the slit width *L*? For *L* > 3 nm, where the dielectric constant is found to saturate in the center of the channel, this dielectric constant can be calculated directly from the simulations by averaging over the central part of the simulation box as indicated by the orange rectangle in Figure 6(B). The orange squares in Figure 6(C) show that the effective dielectric constant in the center of the water slab for 3 < *L* < 10 nm is equal to the bulk value of ε_*bulk*_ = 70. Clearly, inserting ε_⊥_^*eff*^ = ε_*bulk*_ into eq 12 and using the definition of the dielectric dividing surface, eq 8, we find *L*_⊥_^*eff*^ = *L* – 2λ_ε_, with λ_ε_ = 0.15 nm being the distance between the graphene sheet and the dielectric dividing surface, see Figure 6(B). Using this value for *L*_⊥_^*eff*^, we can also calculate ε_⊥_^*eff*^ for *L* < 3 nm from the simulated profiles,^131^ showing a decrease only for *L* < 1.4 nm (Figure 6(C), green diamonds). The second question we may ask is what is the effective dielectric constant of the medium between two electrodes? This is the value measured in experiments,^143,145^ and it is equal to ε_⊥_^*eff*^ if *L*_⊥_^*eff*^ is set equal to the distance between the electrodes. Excellent agreement with the experimental results is obtained for *L*_⊥_^*eff*^ = *L* + 0.3 nm (Figure 6(C), blue diamonds). To calculate the blue curve in Figure 6(C) for values of *L* exceeding 3 nm, the dielectric profiles have been constructed by extending the 3 nm profile with a region of ε_⊥_^–1^ = 1/70 in the center. Since we would expect *L*_⊥_^*eff*^ = *L* if the electrodes are located at the position of the simulated graphene sheets, these results suggest a mismatch between the simulated width *L* and the width *L*_⊥_^*eff*^ fitting the experiments corresponding to the size of one or two layers of water.

In summary, FF MD simulations indicate that the dielectric constant in the interior of water confined in a slit is equal to the bulk dielectric constant down to a slit width of *L* = 1.4 nm. FF MD simulations and experiments agree that the effective dielectric constant between two electrodes exhibits a decrease over much larger length scales, but to obtain quantitative agreement for a graphene slit we have to assume that the effective slit width used in the analysis of the experiments is 0.3 nm wider than the simulated slit width. This slight disagreement could be caused, for example, by incomplete filling or the presence of small amounts of low-dielectric solutes near the electrodes in the experiments. Alternatively, we cannot exclude force field issues in the FF MD modeling as a possible cause.

### 3.4 Viscosity and Surface Slip

The hydrodynamic properties of the electric double layer are closely related to the density and the electrostatic structure discussed in the previous sections. Because of the local density variations, the modified hydrogen bond structure and the molecular orientation, the local effective viscosity in the interfacial layer is expected to deviate from the bulk value.^54^ Direct measurements of friction forces show that the interfacial effective viscosity strongly depends on the hydrophilicity of the surface. The interfacial effective viscosity is enhanced compared to the bulk at hydrophilic silica and mica surfaces^146,147^ but not at C and CH_3_-terminated surfaces.^146^ Also ultrasonic measurements show an enhanced interfacial effective viscosity at hydrophilic Al_2_O_3_ surfaces^66^ and a reduced effective viscosity at hydrophobic alkane–water interfaces.^148^ Conflicting results have been reported for the vapor/water interface.^149−151^

The effects of different interface types on the local effective viscosity profile have been reproduced in FF MD simulations.^59,68,132,153^ In the simulations, velocity profiles *u*_∥_(*z*) are generated in a fluid slab between two surfaces by either shearing the surfaces or, in the presence of ions in the fluid, by applying a parallel electric field. The Stokes equation for flow along a planar surface, using lateral invariance, reads
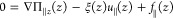
13with Π(*z*) being the shear stress tensor, ξ(*z*) being the surface friction coefficient and *f*_∥_(*z*) being the external body force density. Before atomistic molecular dynamics simulations were available, the surface friction was extracted from solvent-implicit simulations,^154^ yet because molecular dynamics simulations show that the surface friction does not modify the effective viscosity profile significantly,^152^ we set ξ(*z*) = 0 in the following.

**Figure 7:**
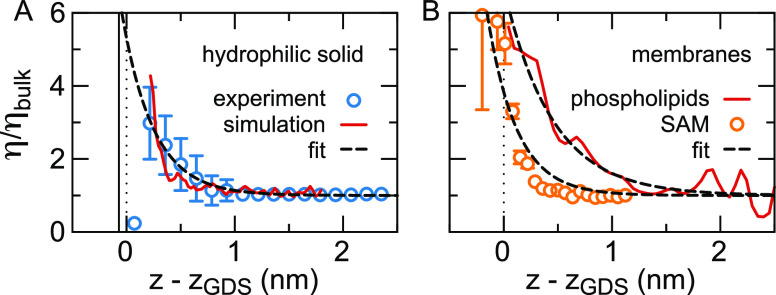
Effective viscosity profile at uncharged hydrophilic surfaces. (A) Simulations of an OH terminated diamond surface in contact with pure SPC/E water, which has η_*bulk*_ = 0.64 mPa·s,^59^ compared to the experimental effective viscosity profile at Al_2_O_3_.^66^ (B) Simulations of a zwitterionic POPC membrane in contact with a 0.44 M NaCl solution (CHARMM 36 membranes and ions with TIP3P water)^132^ and a self-assembled monolayer (SAM) of alcohol chains in pure water (GROMOS 53A6 with SPC/E water) at a separation of 4.27 nm.^152^ The bulk viscosity of the TIP3P water model is η_*bulk*_ = 0.321 mPa·s. The simulated profiles have been fit using η/η_*bulk*_ = *C* exp(−*z*/λ_η_) + 1, with *C* being a constant, giving λ_η_ = 0.29 nm at the hydrophilic solid, λ_η_ = 0.27 nm at the SAM and λ_η_ = 0.39 nm at the phospholipid bilayer membrane.

In the shearing simulations, forces *F*_∥_ and −*F*_∥_ are applied to two solid surfaces, each with surface area *A*, to create a shear profile in the fluid in between. To define the local effective viscosity profile, we start from the linear response relation for the nonlocal inverse viscous response function η_*nl*_^–1^(*z*,*z*^′^),
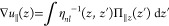
14neglecting any possible dependence on the lateral coordinates and time. Integrating eq 13 once over *z* we find
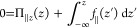
15If the surface–fluid interactions act over a vanishingly small range, the second term on the right-hand side of eq 15 equals *F*_∥_/*A* for *z* inside the fluid, which means that Π_∥*z*_(*z*) is independent of *z*.^152^ Inserting this into eq 14 leads to the definition of the local viscous response function η(*z*),
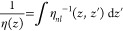
16The Stokes equation for the shear simulations becomes
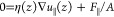
17In Figure 7(A), we show simulation results for η(*z*) at the hydrophilic diamond surface, modeled by an OH-terminated diamond (solid line).^59^ The effective viscosity profiles are normalized by the bulk viscosity η_*bulk*_. The effective viscosity increases steeply at the surface, reaching over four times the bulk value, in agreement with experiments (symbols).^66^ A similar profile is found in shear simulations of a self-assembled monolayer (SAM) of alcohol chains, shown as symbols in Figure 7(B).^152^ Fitting a heuristic exponential function to the simulated effective viscosity profile gives a characteristic length scale of λ_η_ = 0.29 nm at the solid surface and λ_η_ = 0.27 nm at the SAM, equivalent to about one molecular diameter.

As an alternative to shearing, the viscosity can be calculated from electrokinetic flow. In the presence of surface charges or preferentially adsorbing ions, a tangential electric field *E*_∥_ induces an electrokinetic flow profile *u*_∥_(*z*). Combining the Poisson and Stokes equations and the definition of the local effective viscosity in eq 16,^132,141^ the effective viscosity profile is calculated from
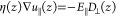
18which has been applied to simulations of a 0.44 M NaCl solution at a phospholipid membrane to calculate η(*z*).^132^ As shown in Figure 7(B), the effective viscosity at the lipid membrane shows a similarly steep increase as the viscosity at the hydrophilic diamond surface. Fitting the simulation data with an exponential function yields a characteristic length scale of λ_η_ = 0.39 nm, slightly larger than at the solid surface, which we attribute primarily to the more diffuse nature of the water/membrane interface.

**Figure 8:**
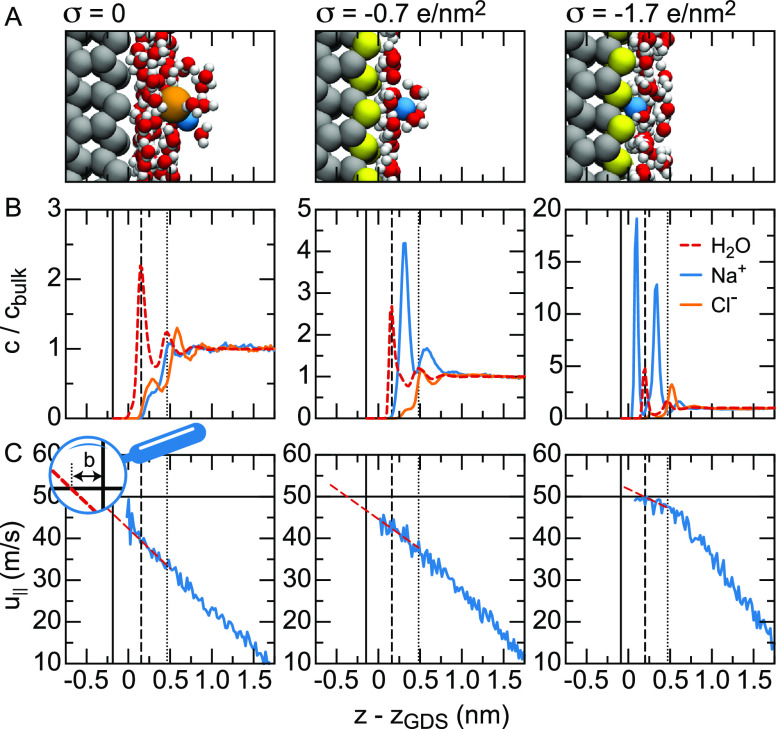
Density of water and ions and velocity profiles at hydrophobic, nonpolar silicon surfaces with different surface charge densities σ.^68^ The bulk salt concentration equals 1.7 M. (A) Simulation snapshots; chloride ions are shown in orange, sodium ions in blue, uncharged silicon atoms in gray and charged silicon in yellow. Not all water molecules are shown. (B) Densities of sodium (blue), chloride (orange) and water oxygen (red) normalized by their bulk densities. The solid vertical lines denote the position of the surface atoms, the dashed lines denote the boundary of the inner Helmholtz layer corresponding to the position of the first water peak, and the dotted lines denote the outer boundary of the outer Helmholtz layer corresponding to the second water peak. (C) Electrolyte velocity profiles *u*_∥_(*z*) (blue) in shear simulations with a surface velocity of 50 m/s. In red, we show the linear extrapolation of the velocity profile in the outer Helmholtz layer.

At hydrophobic surfaces, the behavior is more complicated. Instead of an enhanced interfacial density profile, the interface at hydrophobic surfaces exhibits a density gap,^65,140^ leading to reduced friction.^153,155,156^ The effective viscosity at a hydrophobic surface consisting of a regular array of silicon atoms is calculated using FF MD simulations by shearing a layer of SPC/E water between two surfaces, see Figure 8(A). The surface charge density of the surfaces is increased from zero to σ = −2*e*/nm^2^. The density profiles of water and ions for three different values of σ are shown in Figure 8(B). At low and moderate surface charge densities (|σ| < 1*e*/nm^2^^68^), the first peak of counterions is located between the first two peaks of the water density, which means that the ions are fully hydrated. Therefore, we define the region between the first and second water peaks as the outer Helmholtz layer. At high surface charge density (|σ| > 1*e*/nm^2^), counterions start accumulating between the surface and the first water peak, meaning that the ions have lost a part of their hydration shell. We define the region between the surface and the first water peak as the inner Helmholtz layer. The region outside the Helmholtz layers is defined as the diffuse layer.

### 3.5 Box Profile of the Effective Viscosity

**Figure 9:**
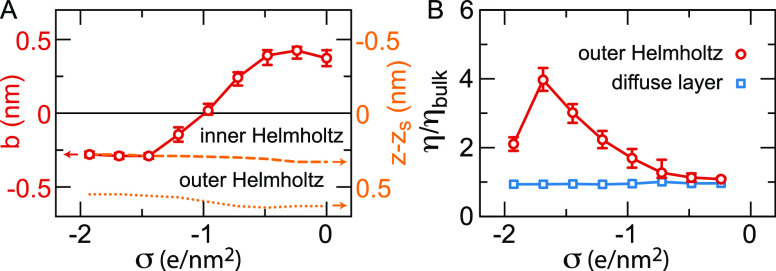
Slip length and effective viscosity at a hydrophobic surface as a function of the surface charge density σ.^68^ (A) Slip length *b* (red symbols, left axis) calculated from the extrapolation of the shear velocity in the outer Helmholtz layer, see Figure 8. Also shown are the positions of the first and second water density peaks with respect to the surface position *z*_*s*_ (orange lines, right axis), defining the boundaries of the inner and outer Helmholtz layers. When the slip length is negative, *b* defines the position of the no-slip plane, coinciding with the boundary of the inner Helmholtz layer for high |σ|. (B) Viscosity of the outer Helmholtz layer and the diffuse layer as a function of the surface charge density.

Figure 8(C) shows a portion of the velocity profiles *u*_∥_(*z*) between two nonpolar silicon surfaces at a separation of 4.84 nm moving in the opposite direction with velocities of *u*_∥_^*s*^ = ±50 m/s.^68^ Below a surface charge density of about |σ| = 1*e*/nm^2^, the hydrodynamics near the interface are characterized by slip along the surface, violating the no-slip boundary condition, as observed previously for hydrophobic surfaces.^157^ The hydrodynamic boundary condition can be modeled effectively using a slip length *b*,
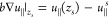
19where *z*_*s*_ refers to the surface position, indicated by solid black lines in Figure 8(B–C). Graphically, this procedure comes down to linearly extrapolating the shear velocity profile near the interface to *u*_∥_^*s*^, see Figure 8(C). Because, when populated, the inner Helmholtz layer is hydrodynamically stagnant, as we will show later, we extrapolate the velocity profile in the outer Helmholtz layer instead to calculate *b*. The extrapolation provides both the slip length and the average effective viscosity of the outer Helmholtz layer. In Figure 9(A) we show *b* as a function of σ together with the position of the surface and water peak positions that define the inner and outer Helmholtz layers. The slip length is positive at the uncharged surface, as confirmed in experiments at hydrophobic uncharged surfaces.^158,159^ The behavior at the uncharged silicon surface shown here is similar to the behavior at the uncharged diamond surface.^59,153^ With increasing magnitude of the surface charge density, the slip length decreases, as observed in simulations previously,^160^ eventually turning negative. A negative slip length means that a layer of fluid is rigidly stuck to the surface, and the no-slip boundary condition moves from *z* = *z*_*s*_ to *z* = *z*_*s*_ – *b*. As soon as the space between the surface and the first water peak is populated with ions, which is the case for |σ| > 1*e*/nm^2^, the no-slip boundary condition is located at or near the boundary between the inner and outer Helmholtz layers, see Figure 9(A), showing that the inner Helmholtz layer is indeed hydrodynamically stagnant.

Fitting the remaining velocity profile (ensuring continuity at the boundary between the diffuse layer and the outer Helmholtz layer) also provides the effective viscosity of the diffuse layer. Figure 9(B) shows the effective viscosity of the outer Helmholtz layer and the diffuse layer.^68^ Independent of the surface charge density, the effective viscosity of the diffuse layer remains equal to the bulk viscosity η_*bulk*_. In contrast, the effective viscosity of the outer Helmholtz layer increases with increasing |σ|, even though a decrease is observed at very high |σ| because of the redistribution of ions from the outer to the inner Helmholtz layer. This leads to the definition of a box profile for the effective viscosity,
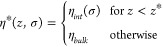
20which together with the boundary condition of eq 19 determines the flow profile. For the silicon surface, z* is located at the outer boundary of the outer Helmholtz layer and η_*int*_ equals the effective viscosity of the outer Helmholtz layer. By fitting the parameters, the model can equally well be used to approximate the effective viscosity profiles at hydrophilic surfaces shown in Figure 7. A box profile for the effective viscosity has been used successfully to model the electrokinetic mobility of charged colloids as a function of their surface charge density,^137,139^ see section 4.6.

In summary, the effective viscosity at hydrophilic surfaces is enhanced regardless of surface charge density. In contrast, Figures 8 and 9 show that the hydrodynamics of hydrophobic surfaces changes from classical slipping behavior for uncharged surfaces, where the effective viscosity remains equal to the bulk value while the fluid slips along the wall via a combination of an enhanced interfacial effective viscosity and reduced surface slip at intermediate surface charge density, to an enhanced interfacial effective viscosity in combination with a stagnant inner Helmholtz layer at high surface charge density.

## 4 Ions at Charged and Uncharged Surfaces

To model ion distributions near interfaces, we start by considering the free energy of a single ion as a function of the distance to an uncharged surface, referred to as the potential of mean force Δ*F*(*z*). Afterward, we discuss how to incorporate the potential of mean force into mean-field theories to model single ions at charged interfaces and ion solutions at finite concentration.

### 4.1 Single Ions at Uncharged Interfaces

**Figure 10:**
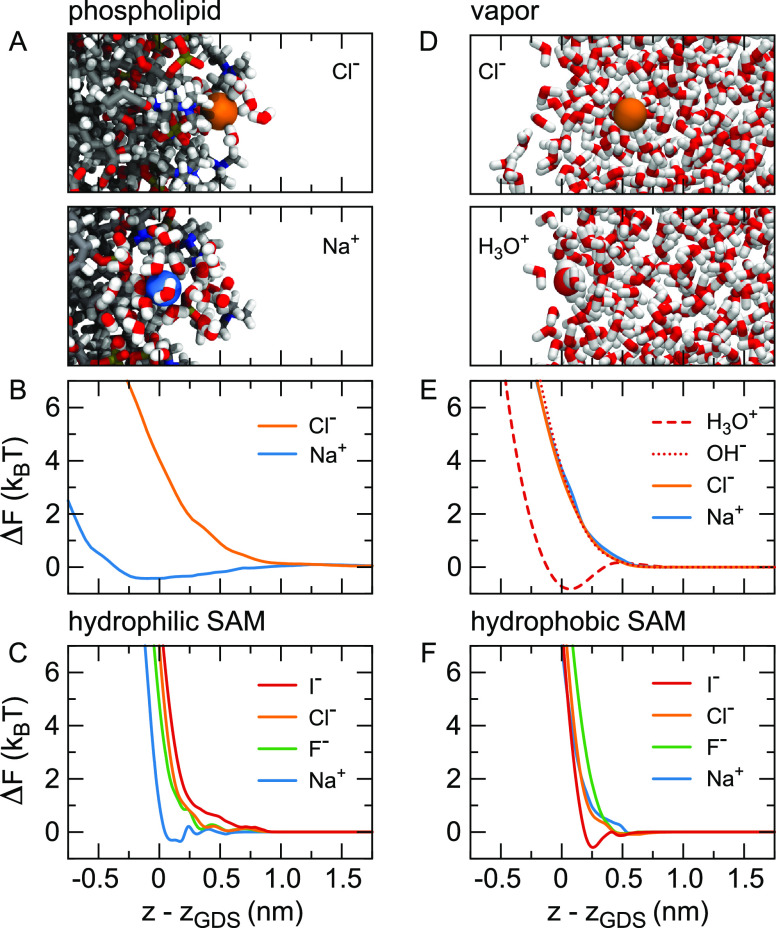
Ionic potentials of mean force. (A) Snapshots of a Cl^–^ (orange) and a Na^+^ (blue) ion at a lipid membrane together with their hydration shells. Other water molecules are not shown. Na^+^ is located near the minimum of the potential of mean force, and Cl^–^ is located at 0.5 nm from the GDS, where the PMF is about 1*k*_B_*T*. Nitrogen atoms are shown in blue, and phosphor atoms are shown in bronze. (B) Corresponding potentials of mean force.^132^ (C) Potentials of mean force at a hydrophilic self-assembled monolayer.^161^ (D) Snapshot of Cl^–^ (orange) and H_3_O^+^ (red and white) at the vapor/water interface. (E) Corresponding potentials of mean force.^77^ (F) Potentials of mean force at hydrophobic self-assembled monolayers.^161^

The potential of mean force Δ*F*(*z*) is calculated from FF-based MD simulations using either umbrella sampling, where the free energy is calculated from the position distribution while restraining the ion at varying distances from the surface, or by thermodynamic integration, where the energy of inserting the ion at a given position is calculated.^117^ In Figure 10 we show the potentials of mean force for a number of different ions at both hydrophilic and hydrophobic surfaces. The potentials of mean force are strongly ion- and surface-specific, but some trends can be clearly distinguished. Comparing Figures 10(B–C) and (E–F) shows that a small ion like sodium adsorbs at hydrophilic interfaces but not at hydrophobic ones. A large ion like iodide, in contrast, adsorbs at the hydrophobic SAM, consisting of a restrained grid of alkanes,^162^ but not at the hydrophilic SAM, consisting of a restrained grid of alcohol chains. Chloride is repelled from all interfaces. The hydronium ion, H_3_O^+^, shows atypical behavior, adsorbing relatively strongly at the vapor/water interface. The adsorption has been attributed to the favorable orientation of its dipole in the interfacial electric field.^77^ For monatomic ions, adsorption at the vapor/water interface has only been observed in simulations of iodide.^163^

**Figure 11:**
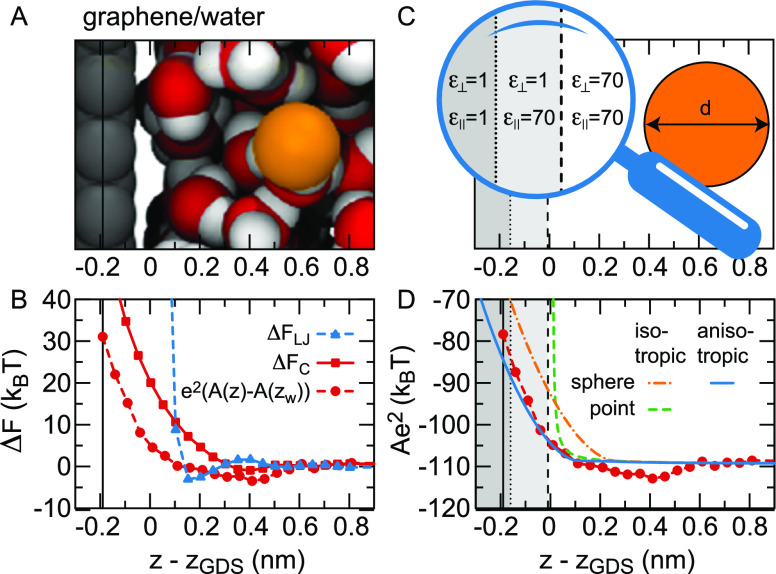
Decomposition of the potential of mean force.^117^ (A) Snapshot of a chloride ion at an uncharged graphene surface. (B) Potential of mean force split into Coulomb and Lennard-Jones terms. We also show the linear Coulomb term *A*(*z*)*e*^2^ relative to its bulk value *A*(*z*_*w*_)*e*^2^. (C) Anisotropic model of the dielectric environment. (D) Linear Coulomb term *A*(*z*)*e*^2^ calculated for chloride from simulations (red symbols), for a point charge (green), and for a sphere with diameter *d* = 0.508 nm and surface charge density σ = −*e*/(π*d*^2^) in an isotropic (orange) and an anisotropic (blue) dielectric environment.

To better understand the mechanisms behind these potentials of mean force, we use thermodynamic integration to split the potentials into different contributions according to
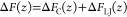
21with Δ*F*_LJ_(*z*) stemming from the creation of a neutral Lennard-Jones cavity and Δ*F*_C_(*z*) from charging that cavity. Note that Δ*F*_LJ_ contains Lennard-Jones as well as Coulombic interactions, the latter originating in the necessary rearrangement of the water, just like Δ*F*_C_ contains both Lennard-Jones and Coulomb contributions. We study the case of a chloride ion at a graphene surface as shown in Figure 11(A). As can be seen in Figure 11(B), the dominant part of the repulsion at the graphene interface comes from Δ*F*_LJ_(*z*). Apart from the strong repulsion, Δ*F*_LJ_(*z*) shows a pronounced minimum (Figure 11(B), blue broken line and triangles). By splitting Δ*F*_LJ_(*z*) further into ion–water and ion–wall contributions (see supplement of ref (117)), it can be seen that the minimum in Δ*F*_LJ_(*z*) is caused by the ion–wall interaction. Closer to the interface, the ion–water contribution also becomes negative due to the vanishing water density, but this contribution is negligible compared to the repulsive ion–wall interaction.^117^ Although Δ*F*_LJ_(*z*), the energy required to create a Lennard-Jones cavity, is a different quantity than the energy associated with the Lennard-Jones interactions between an ion an its environment, a region of attractive Lennard-Jones interaction is generally expected. Nevertheless, the Lennard-Jones interaction between an ion and a soft self-assembled monolayer, obtained by splitting the potential of mean force into Coulomb and Lennard-Jones contributions, has previously been found to be entirely repulsive.^162^

The energy associated with charging the cavity, Δ*F*_C_(*z*) (Figure 11(B), red solid line and squares), is largely repulsive but dominates only in a very small region near the surface. To analyze the Coulomb contribution in more detail, we express it as an expansion in terms of the ion charge *q* according to eq 4, as discussed in section 2.3. Assuming that the water is a linear homogeneous dielectric medium, the repulsion from a dielectric interface scales with *q*^2^. Therefore, to demonstrate how the simulated Coulomb contribution is related to the linear dielectric response theory, we interpret the term *A*(*z*)*q*^2^ from eq 4 using different models for the dielectric environment. To keep the models analytically tractable, we describe the dielectric medium using the box models based on *z*_⊥_^DDS^ and *z*_∥_^DDS^ defined in eq 8. First, we show the free energy for a point charge in an isotropic dielectric with ε_⊥_ = ε_∥_ = 1 for *z* < *z*_GDS_ and ε_⊥_ = ε_∥_ = 70 otherwise, showing the well-known divergence at *z* = *z*_GDS_ (Figure 11(D), green broken line). Second, the energy is calculated for a sphere with a finite diameter, the value of which is determined from the Born energy, i.e., the solvation free energy of a charged sphere in a homogeneous dielectric medium. Since the Born energy is proportional to the square of the charge, we can equate it to *A*(*z*_*w*_)*e*^2^, from which the ion diameter *d* follows as
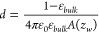
22giving *d* = 0.508 nm.^117^ The ion is modeled by a sphere with surface charge density σ = −*e*/(π*d*^2^) and dielectric constant inside equal to the dielectric environment outside. The free energy for this finite-sized sphere in the same isotropic dielectric environment as the one used for the point charge (ε_⊥_ = ε_∥_ = 1 for *z* < *z*_GDS_ and ε_⊥_ = ε_∥_ = 70 otherwise) does not diverge (Figure 11(D), orange dash–dotted line).^117,164^ However, it also fails to reproduce the simulated values of *A*(*z*)*e*^2^. Third, the free energy is calculated for a finite-sized sphere in an anisotropic dielectric medium. The fact that *z*_⊥_^DDS^ and *z*_∥_^DDS^ generally differ gives rise to three different regions as shown in Figure 11(C), viz., a region with ε_⊥_ = ε_∥_ = 1, comprising the solid, an anisotropic region with ε_⊥_ = 1 and ε_∥_ = 70, and a region with ε_⊥_ = ε_∥_ = 70, comprising the bulk fluid. Calculating the free energy in this anisotropic dielectric environment yields excellent agreement with the simulations, see the blue solid line in Figure 11(D). This calculation shows that the anisotropic local dielectric environment has a strong effect on the electrostatic part of the potential of mean force, providing a source of surface specificity through *z*_⊥_^DDS^ and *z*_∥_^DDS^, as well as ion specificity through the effective ion diameter *d*.

In the calculations above, it is assumed that the dielectric profile of the environment, which includes the space inside the ion, is unaffected by the presence of the ion. The resulting free energy is indistinguishable from the free energy of a nonpolarizable sphere, having ε_⊥_ = ε_∥_ = 1 inside.^117^ If instead the ions are modeled as being perfectly polarizable (ε_⊥_ → *∞*, ε_∥_ → *∞*), the image charge repulsion in the interfacial region is reduced, allowing ions to move closer to the interface.^165,166^ The reduction of the image charge repulsion has been confirmed by molecular dynamics simulations with polarizable ion force fields.^59,167^ Note, however, that the ion’s surface excess at the vapor/water interface, including adsorption of hydronium ions, can be quantitatively reproduced by well-optimized force fields without explicit polarizability.^77^ In that case, the ion’s polarizability is effectively incorporated in the Lennard-Jones part of the interaction potential.

### 4.2 Box Profile of the Potential of Mean Force

To simplify the inclusion of the potential of mean force into the Poisson–Boltzmann equation, we define a box model for the ion–surface interaction potential μ_*i*_(*z*),
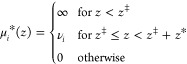
23The adsorption strength ν_*i*_ can be extracted from MD simulations for every ion type *i* by equating the surface excess relative to *z*^⧧^ predicted by eq 23 to the surface excess predicted by the potential of mean force,
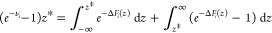
24At the vapor/water interface, the Gibbs dividing surface is a natural choice for the position *z*^⧧^. Other choices are also possible, such as *z*^⧧^ = *z*_*s*_ at solid surfaces. The range z* of the potential is a free parameter, for which we choose z* = 0.5 nm at the vapor/water interface based on the MD result that the potentials of mean force start to deviate from zero at that position, see Figure 10(E). The expression in eq 24 is known as the Henry adsorption coefficient.^168^

### 4.3 Extended Poisson–Boltzmann Equations

At charged interfaces and at finite salt concentrations, interactions with other ions and with the surface charge have to be taken into account, for which a modified mean-field theory has been developed. A system with ions has a nonzero monopole density *P*_0_(*z*), which is the free charge in the multipole expansion shown for the polarization in eq 3, and therefore produces a spatially varying displacement field *D*_⊥_(*z*). In this case the local expression for the inverse dielectric profile ε_⊥_^–1^(*z*) in eq 10 is not strictly valid. However, if the variation of the displacement field is slow compared to the variation of the electric field, we can use the locality approximation
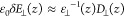
25where we additionally assume that changes from the situation with *P*_0_ = 0 are small, equating the local change in the displacement field to the displacement field itself. Equation 25 is valid at low surface charge density or low salt concentration.^10,127^ We use ∇δψ(*z*) = −δ*E*_⊥_(*z*), with δψ(*z*) being the excess electrostatic potential caused by the field *D*_⊥_(*z*), see Figure 1, and ∇*D*_⊥_(*z*) = *P*_0_(*z*), with *P*_0_(*z*) being the ionic charge density. Taking the derivative of eq 25, we arrive at the extended Poisson equation
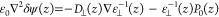
26Multiplying eq 26 by ε_⊥_^–1^(*z*) and inserting eq 25, we arrive at
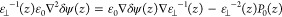
27For a solution of monovalent ions, the free charge density is calculated from the ionic densities *c*_+_(*z*) and *c*_–_(*z*),
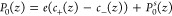
28with *e* being the absolute value of the elementary charge and *P*_0_^*s*^(*z*) being the charge density on the surface. For soft surfaces and for surfaces with a porous surface layer, the surface charge density has a finite spatial extension, which can be taken into account through the surface charge density profile *P*_0_^*s*^(*z*). For solid, nonporous surfaces, the surface charge density is typically taken into account as a boundary condition, using
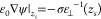
29with σ being the surface charge density. To ensure that the ion density at the surface does not exceed its physical limit set by the ionic volume, we include a Fermionic steric interaction to calculate the ion density profiles from the unrestricted ionic densities  and ,^11−15^
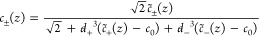
30with *c*_0_ being the bulk salt concentration and *d̵*_+_ and *d̵*_–_ being the steric diameters of positive and negative ions, respectively. Note that these diameters are typically different from the dielectric diameter *d* introduced in section 4.1.^108,169^ The denominator in eq 30 restricts the maximum density *c*_±_(*z*) to , which is the maximum density of close-packed (face-centered cubic or hexagonal close-packed) spheres of diameter *d̵*_±_. For small ions at low salt concentration or at vanishing surface charge density, the steric interaction does not affect the ion density profiles.^170^ The unrestricted ionic densities  and  obey the Boltzmann distribution
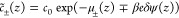
31with β = 1/(*k*_B_*T*) being the inverse thermal energy and μ_+_(*z*) and μ_–_(*z*) being the nonelectrostatic contributions to the potential of the positive and negative ions, respectively. Combining eqs 27, 28, 30 and 31 yields the extended Poisson–Boltzmann equation that we will use in the following sections.

### 4.4 Charged Interfaces with Counterions Only

Before solving the extended Poisson–Boltzmann equation, we have to determine what to use for the nonelectrostatic potential μ_±_(*z*). In particular, an important contribution to the interaction of an ion with an interface is the image charge repulsion, which is of Coulombic origin. The image charge interaction is included in the potential of mean force Δ*F*_±_(*z*), as shown in Figures 10 and 11, but not in the Poisson–Boltzmann equation given by eqs 27, 28, 30 and 31. At interfaces where the image charge repulsion dominates the ion–surface interaction, it needs to be incorporated one way or another, but strictly speaking it is inconsistent to include the image charge potential as a nonelectrostatic contribution. At solid surfaces, the Lennard-Jones contribution dominates the potential of mean force, so we could use the complete PMF Δ*F*_±_(*z*) as an approximation of μ_±_(*z*). However, if image-charge effects are included in the PMF, they are included only on the single-particle level while two-body and higher-order correlation effects are neglected. In general, the effect of the missing image charge potential, as well as the effects of the neglect of correlation effects, nonideal electrolyte activity and concentration dependent dielectric decrement, are complex and partially cancel each other.^171^ Therefore, considering the level of approximation used in the extended Poisson–Boltzmann equation, we deem the introduction of a heuristic image charge potential in the Poisson–Boltzmann equation, as we do below, as justified. Such a heuristic image charge interaction has been used to accurately reproduce Monte Carlo simulations of ion-specific effects at interfaces.^171^

**Figure 12:**
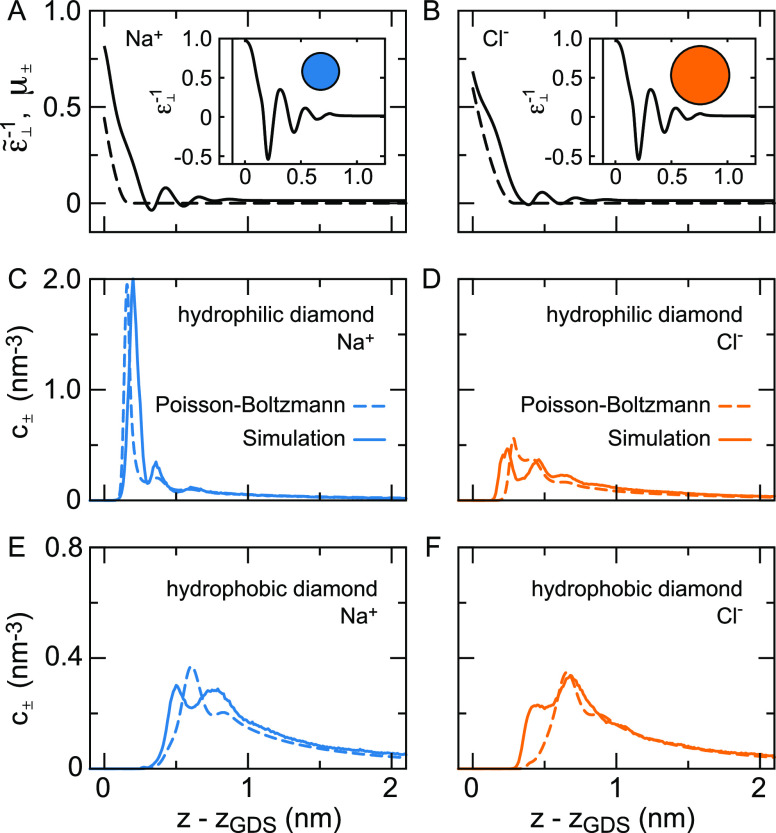
Input and results of the extended Poisson–Boltzmann calculation.^59^ (A–B) The effective dielectric profile  (solid black line, eq 33) and the nonelectrostatic potential μ_±_(*z*) (broken black line, multiplied by 10^–2^, eq 32) are constructed by convoluting the dielectric profile ε_⊥_^–1^ (eq 33) and a planar solid wall (eq 32) with a spherical ion, as illustrated in the insets. The diameter of the sodium ion is *d*_+_ = 0.330 nm for the dielectric convolution and *d̵*_+_ = 0.282 nm for the ion–wall interaction; for chloride the diameters are *d*_–_ = 0.446 nm and *d̵*_–_ = 0.402 nm. The dielectric profiles used for the convolution, shown in the insets of panels (A) and (B) for the hydrophilic diamond, are identical. (C–F) The result of the extended Poisson–Boltzmann calculation (broken lines, eqs 27, 28, 30 and (31)) compared to the simulation result (solid lines) in the limit of vanishing bulk salt concentration at different diamond surfaces with a surface charge density of σ = 0.3*e* nm^–2^ for the chloride simulation and σ = −0.3*e* nm^–1^ for the sodium simulation. Results at the hydrophilic diamond surface are shown in (C–D) and at the hydrophobic diamond surface in (E–F).

Based on the interactions of a sphere with a wall and with a water phase, alternative models for the nonelectrostatic potential without image charge contribution have been introduced.^59,67^ At a solid surface, we use a model aimed at capturing the basic nonelectrostatic features of the Lennard-Jones-dominated PMF of monatomic ions by using the convolution of a sphere with a solid wall with interaction strength *B*_±_,
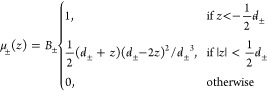
32with *z* being the distance from the surface (located at *z*_*s*_ = 0) and *d̵*_±_ being the steric ionic diameter. For the results shown in Figure 12(A–B) (broken lines), the Lennard-Jones radii of the simulated ions have been used for *d̵*_±_ and *B*_±_ = 200.^59^

In the linear response regime, nonpolarizable ions in inhomogeneous dielectrics can be treated as point charges in an effective dielectric profile given by the convolution of the dielectric profile with the normalized surface area of the ion.^172^ Accordingly, the dielectric profiles ε_⊥_^–1^(*z*) are convoluted with the normalized surface area of a sphere of diameter *d*_±_, see Figure 12(A–B),
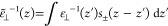
33with the normalized atomic surface area density being given by
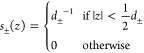
34The dielectric diameter *d*_±_ is estimated from the ionic solvation free energy Δ*F*_±_(*z*) as^108,169^
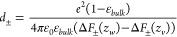
35using the complete solvation free energy for convenience, instead of only the quadratic Coulombic part as in eq 22. Convolution according to eqs 33 and 34 is identical to calculating a running average over *d*_±_. Using the solvation free energy Δ*F*_±_(*z*_*w*_) – Δ*F*_±_(*z*_*v*_) with respect to which the MD force fields are optimized,^173^ we find *d*_–_ = 0.446 nm for Cl^–^ and *d*_+_ = 0.330 nm for Na^+^. Note that the value for Cl^–^ differs by 12% from the value used in section 4.1, where only the *q*^2^ dependent contribution of the Coulombic part of Δ*F*_±_(*z*) has been used. Apart from high positive electric fields at hydrophobic surfaces, external electric fields do not affect the dielectric profile much,^108,142^ as will be discussed in more detail in section 4.6. Therefore, we use the dielectric profile determined at zero external electric field.

In Figure 12(C–F) we show the densities of Na^+^ and Cl^–^ at a hydrophilic and a hydrophobic diamond surface with surface charge density σ = ±0.3*e*/nm^2^ calculated using the extended Poisson–Boltzmann equation (broken lines). The calculations are compared to the results of FF-based MD simulations with only counterions present. The agreement is reasonably good in all cases. In particular, the strong adsorption of sodium onto hydrophilic surfaces, observed already in the PMFs (Figure 10), is faithfully reproduced. This calculation shows that the deviations of the ion densities at charged solid surfaces from the Gouy–Chapman model, including ion-specific effects, can be modeled by a combination of the dielectric profile and the steric repulsion of a sphere from a solid wall. Specifically, the low-dielectric region near the interface enhances the electrostatic interactions, causing extra attraction of ions that are small enough to enter this region.

### 4.5 Ion Adsorption at Finite Bulk Concentration

Ultimately, we test the extended Poisson–Boltzmann equation in the case of finite salt concentrations at a zwitterionic surface. At phospholipid membranes, ion-specific interactions cause adsorption of sodium over chloride, which is modeled using the extended Poisson–Boltzmann equation from section 4.3. In contrast to solid surfaces, the Lennard-Jones repulsion does not dominate the PMF. Instead, the interaction of the lipids with the lipid headgroup partial charges and the image charge interaction are primarily responsible for the repulsion of the ions from the lipid phase. Therefore, the complete ionic potential of mean force shown in Figure 10(B) is used for μ_±_(*z*). This procedure heuristically introduces the image charge repulsion into the Poisson–Boltzmann equation, as discussed in section 4.4. We set *P*_0_^*s*^(*z*) = 0 in eq 28, because effects due to the lipid charge distribution are included in the ionic PMF, and use the dielectric profile from Figure 5(E). Because the dielectric profile at the phospholipid membrane essentially consists of a smooth decrease over about 1 nm, exceeding the ion size, it has little influence on the calculated ion densities.

**Figure 13:**
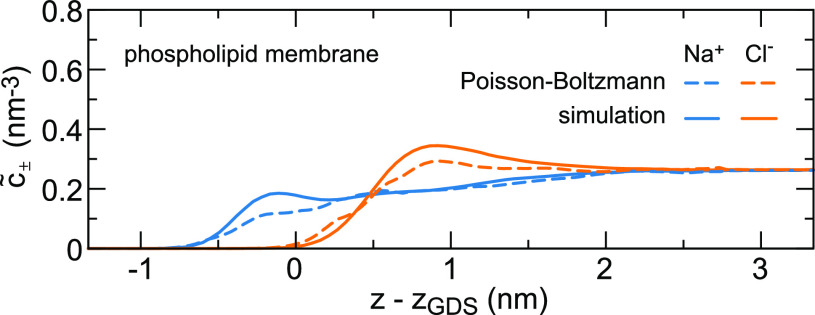
Sodium and chloride concentration profiles resulting from the extended Poisson–Boltzmann calculation (broken lines) compared to the simulation result (solid lines) at a POPC lipid membrane at a bulk salt concentration of 0.44 M NaCl (CHARMM 36 with TIP3P). The dielectric profile from Figure 5(E) is used for ε_⊥_^–1^(*z*), and the potential of mean force shown in Figure 10(B) is used for the nonelectrostatic potential μ_±_(*z*).^132^

The ion densities at the lipid membrane are shown in Figure 13. Clearly, the extended Poisson–Boltzmann equation is capable of reproducing the densities of Na^+^ and Cl^–^ at zwitterionic surfaces, including the ion-specific preferential adsorption of sodium.

### 4.6 Effect of Ions and Electric Fields on the Dielectric and Viscous Properties

So far, we have used the approximation that the viscosity and the dielectric properties of the fluid are not disturbed by the local interfacial electric field or the ion concentration, which is a good approximation for double layers at surfaces with low charge densities. Yet close to highly charged interfaces, the electric field is very high, and so is the ion concentration. In bulk, both applied electric fields and added salt have a strong influence on the dielectric constant and the viscosity, and it has been hypothesized that the same effects are important near charged interfaces.^55,174,175^

**Figure 14:**
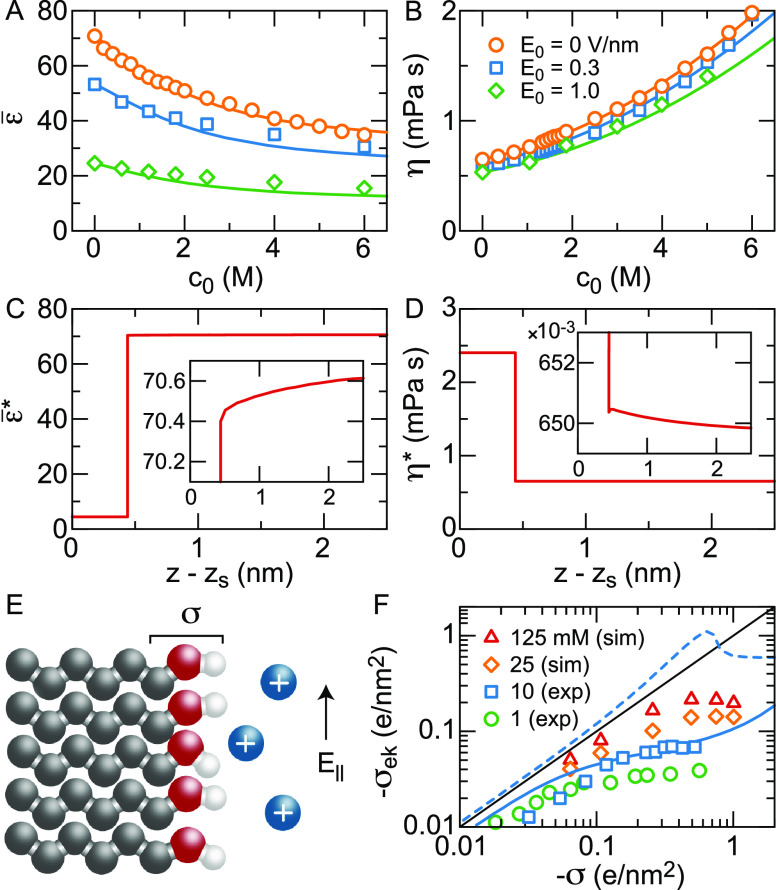
Effects of the ion concentration *c*_0_ and the electric field strength *E*_0_ on the dielectric constant and the viscosity.^142^ (A) The dielectric constant in bulk water from MD simulations (symbols) decreases with increasing *c*_0_ and with increasing *E*_0_. Solid lines denote fits with eq 39. (B) The viscosity of bulk water from MD simulations (symbols) increases with increasing *c*_0_ but decreases slightly with *E*_0_ for fields below 1 V/nm. Solid lines denote fits with eq 42. (C) The dielectric profile and (D) the effective viscosity profile resulting from the solution of the Poisson–Boltzmann equation with the dielectric profile of eq 43 and using the effective viscosity profile of eq 44. The bulk salt concentration is 10 mM. The insets show magnifications of the areas around the dividing surface of the effective viscosity and the dielectric constant. (E) Sketch of a charged decanol surface with counterions used in the electrokinetic simulations. The water is not shown. (F) Electrokinetic surface charge density σ_*ek*_ as a function of bare surface charge density σ in FF-based simulations and experiments (symbols). The lines show the result of the extended Poisson–Boltzmann equation at *c*_0_ = 10 mM with the extended box model using z* = 0 in eqs 43 and 44 (broken blue line) and using values for z*,  and η_*int*_ extracted from MD simulations^139^ (solid blue line). Note that using eqs 43 and 44 with ε̅(0,0) and η(0, 0) for *z* > z* yields results that are indistinguishable from the solid blue line (curves overlap).^142^ The solid straight black line denotes the Stokes–Gouy–Chapman model, where σ_*ek*_ = σ.

The influence of the field and the ion concentration is estimated by calculating the dielectric constant ε(*E*_0_, *c*_0_) and the viscosity η(*E*_0_, *c*_0_) in bulk for different electric fields *E*_0_ and salt concentrations *c*_0_. In eq 10, the dielectric constant ε is defined as the proportionality constant between an infinitesimal increment in the displacement field and an infinitesimal increment in the electric field, which can be calculated from the polarization fluctuations or by applying a finite field.^128^ When including an electric-field dependent dielectric constant in the Poisson equation, however, the relevant quantity is the dielectric difference constant ε̅, defined as the proportionality constant between a finite displacement field and a finite electric field,^142^
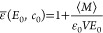
36with *M* being the polarization in the direction of the electric field and *V* being the volume. As can be seen in Figure 14(A), the dielectric difference constant decreases both with increasing salt concentration and with increasing electric field. At vanishing salt concentration, an expression for the dielectric difference constant parallel to the applied electric field has been derived based on the nonlinear dielectric response of a simple dipole in the absence of many-body effects,^176^
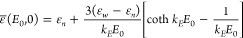
37with ε_*n*_ and *k*_*E*_ being fit parameters and ε_*w*_ being the dielectric constant of pure water. For low electric fields, an expansion of eq 37 yields the quadratic decrease . Equation 37 perfectly fits the data obtained from the FF-based MD simulations.^142^ The fit parameters are *k*_*E*_ ≈ 8.01 nm V^–1^ and ε_*n*_ ≈ 1.80, which is close to the expected ε_*n*_ = 1. The effect of the salt concentration on the dielectric properties is also caused by the electric field, this time emanating from the ions. At vanishing electric field, the dependence of the dielectric constant on the salt concentration is accurately described by the equation^177^
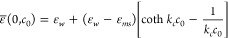
38with ε_*ms*_ being the limiting value of the dielectric constant for very high salt concentrations (molten salt) and *k*_*c*_ being a fit parameter which is related to the excess polarizability α of the ions via *k*_*c*_ = 3α/(ε_*w*_ – ε_*ms*_). Note that . The orange solid curve for *E*_0_ = 0 in Figure 14(A) shows that eq 38 provides an excellent fit to the simulation data. The fit parameters are ε_*ms*_ ≈ 28.5 and α ≈ −12.3 M^–1^, comparing well to the experimental data reported for NaCl (ε_*ms*_ = 27.9, α = −11.59 M^–1^).^171^ To describe the combined effect of the finite *E*_0_ and *c*_0_, the expressions of eqs 38 and 37 are combined using a multiplicative assumption
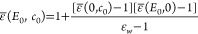
39Equation 39 ensures that  and . The solid curves in Figure 14(A) show that the multiplicative assumption yields an accurate description of .

The dependence of the viscosity η(0, *c*_0_) on the salt concentration *c*_0_ is well described by a second degree polynomial (Figure 14(B), orange line),
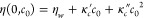
40Note that the asymptotic concentration dependent viscosity at low *c*_0_ is in fact proportional to ,^178^ which becomes significant at low concentrations (*c*_0_ < 0.5 M). At the high concentrations treated here, the square root term can be neglected, but the quadratic term is included on a phenomenological basis instead. The fit parameters are found to be κ_*c*_^′^ = 77.7 × 10^–3^ mPa·s/M and κ_*c*_^″^ = 22.3 × 10^–3^ mPa·s/M^2^. To model the viscosity as a function of electric field strength at vanishing salt concentration, the viscosity is interpolated using a heuristic fit function. At high electric field, the viscosity increases quadratically, and for symmetry reasons the viscosity needs to be a function of even powers of the field *E*_0_. A simple empirical ansatz for η(*E*_0_, 0), which is consistent with these requirements, is given by
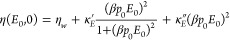
41where κ_*E*_^′^ and κ_*E*_^″^ are fit parameters, β is the inverse thermal energy and *p*_0_ = 0.049*e* nm is the dipole moment of a single water molecule, for which the SPC/E model is used here. Fitting yields κ_*E*_^′^ = −0.190 mPa·s and κ_*E*_^″^ = 8.90 μPa·s for the viscosity component perpendicular to the applied field. Like for the dielectric difference constant, a multiplicative combination of eqs 40 and 41 is used to model the viscosity at finite concentration and electric field,
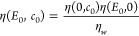
42

The reduced bulk dielectric constant and increased bulk viscosity at high electric field strengths and high ion concentrations are qualitatively consistent with the properties of the interfacial layer at charged surfaces discussed in sections 3.1 and 3.4. To test whether the concentration and electric field dependent response of bulk water is sufficient to describe the interfacial properties, we consider a model where the dielectric and viscous properties of the entire double layer are governed by eqs 39 and 42. These equations are used with μ_±_(*z*) = 0, *d̵*_±_ = 0.3 nm, *P*_0_^*s*^(*z*) = 0 and *c*_0_ = 10 mM, to calculate the electrokinetic surface charge density σ_*ek*_, defined in ref (141), by self-consistently solving the extended Poisson–Boltzmann equation (eqs 27, 28, 30 and 31) and the Stokes equation (eq 18). For the calculation of  when *c*_±_(*z*) ≠ *c*_0_, the approximation is made that anions and cations have the same effect on the dielectric constant, defining the local ion concentration as . A fixed surface charge density σ is used as the boundary condition at the surface, located at *z* = *z*_*s*_ = 0. The calculated σ_*ek*_ is plotted as a broken line in Figure 14(F) as a function of the bare surface charge density σ.^142^ The failure to reproduce the electrokinetic surface charge density of charged TiO_2_ colloids (blue symbols), which has been obtained by measuring their electrophoretic mobility, shows that the dielectric and viscous properties of the double layer are not caused by the effect of the electric field and the ions on the water alone. This observation is consistent with the finding that the modified interfacial properties as shown in Figure 5 are also present at uncharged surfaces and in pure water and that the effect of external electric fields up to 2 V/nm on the interfacial dielectric profiles is negligible.^108^ Therefore, to obtain quantitative agreement with the experiments, we maintain a version of the box models of the interfacial layer introduced in sections 3.2 and 3.5, but we incorporate the dependence on electric field and salt concentration in the remaining space. For the dielectric profile,
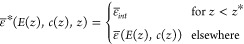
43where *E*(*z*) and *c*(*z*) are determined by self-consistently solving the extended Poisson–Boltzmann equation from section 4.3 with the same settings as previously in this section, but using the inverse of eq 43 for the inverse dielectric profile in eqs 27 and 29. To parametrize the box model, the quotient of the interfacial dielectric constant  and the width of the interfacial layer z* are extracted from FF MD simulations of the dielectric profile.^127^ To achieve quantitative agreement with experimental data, the value of z* is treated as a fit parameter, which simultaneously determines .^139^ Note that this automatically incorporates a possible dependence of z* on, for instance, the surface charge density and the salt concentration, if these affect the interfacial water structure. Similar to eq 43, we extend the box model for the effective viscosity profile with the dependence on the salt concentration and the electric field,^137,141^
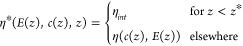
44with η_*int*_ being the interfacial viscosity. For z* we use the same value as in eq 43. We will refer to the model of eqs 43 and 44 as the “extended box model”.

The dielectric and viscous profiles resulting from the self-consistent solution of the Poisson–Boltzmann equation with eq 43 are shown in Figure 14(C–D). Clearly, the influence of the electric field and concentration dependence of  and η*(*E*, *c*, *z*) is very small compared to the effect of the dielectric dividing surface and the box model for the viscosity. This fact is reflected in the electrophoretic surface charge density σ_*ek*_ calculated from the Stokes and extended Poisson–Boltzmann equations using the extended box model, shown as a blue solid line in Figure 14(F). As the comparison with the experimental data (blue squares) shows, the measured electrokinetic mobility of charged colloids is accurately reproduced using the extended box model for the viscosity and the dielectric constant. Surprisingly, the inclusion of the dependence on field strength and concentration makes no difference compared to using only the dielectric and viscous box models (curves overlap).^142^ This means that the box parts of eqs 43 and 44, essentially corresponding to the contribution from the interfacial water structure, dominate the dielectric and viscous properties in the electric double layer. Note that the presence of ions and surface charge can still affect the interfacial water structure, but the bulk-like dependence of the viscosity and dielectric constant on the electric field and the ion concentration can be neglected.

### 4.7 Charged Surfactants

**Figure 15:**
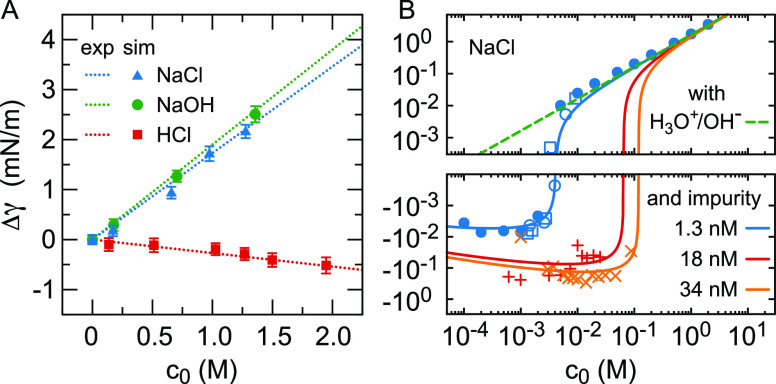
Surface tension change of the vapor/water interface as a function of salt concentration: (A) in the presence of NaCl, NaOH and HCl from experiments (dotted lines) and FF MD simulations (symbols)^77^ and (B) as a function of NaCl concentration in the presence of H_3_O^+^, OH^–^ and surface-active charged impurities. Lines depict the results of eq 48, and symbols denote experimental results. Open squares, bubble pressure (powder);^179^ open circles, bubble pressure (single crystal);^179^ solid circles, capillary rise;^180^ plus signs, Wilhelmy plate (H_2_O);^181^ diagonal crosses, Wilhelmy plate (D_2_O).^181^

Charges on the surface of solutes in aqueous solution can be caused by a chemical reaction, such as deprotonation, but also by adsorption of charged species. In fact, the vapor/water interface also has been found to be negatively charged, which must be caused by the adsorption of charged species. The adsorption causes a change in the surface tension γ according to the Gibbs adsorption isotherm,^182^
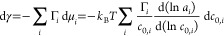
45with μ_*i*_ (without *z*-dependence to distinguish it from the nonelectrostatic potential) being the bulk chemical potential of species *i*. The activity is denoted by *a*_*i*_, and the bulk concentration is denoted by *c*_0,*i*_. The sum in eq 45 is performed over all nonaqueous species, and the surface excess of species *i* is given by
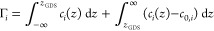
46To express the surface tension in terms of the salt concentration for a salt with valencies υ_+_ and υ_–_, the chemical potential of the salt can be expressed as μ_salt_ = υ_+_μ_+_ + υ_–_μ_–_, with μ_+_ and μ_–_ being the chemical potentials of the positive and negative ions, respectively. The bulk ion concentrations equal *c*_0,+_ = υ_+_*c*_0_ and *c*_0,–_ = υ_–_*c*_0_. Using the mean activity , with υ = υ_+_ + υ_–_, we find μ_salt_ = μ_salt_^0^ + υ*k*_B_*T* ln *a*_salt_, with μ_salt_^0^ being a reference value. Inserting this expression into eq 45 gives
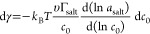
47with υΓ_salt_ = Γ_+_ + Γ_–_. Equation 47 shows that the change in surface tension γ is directly proportional to the change in bulk salt concentration *c*_0_ with a proportionality constant depending linearly on the summed surface excess of cations and anions. The prevalent hypothesis explaining the negative charge on vapor/water and other hydrophobic aqueous interfaces has been the adsorption of OH^–^ ions,^183^ yet OH^–^ adsorption is inconsistent with direct surface tension measurements.^77,184,185^ In particular, the measured surface tension of the vapor/water interface increases more with the addition of NaOH than with the addition of NaCl, see the dotted lines in Figure 15(A). In contrast, the surface tension decreases with the addition of HCl, showing that whereas OH^–^ desorbs from the interface, H_3_O^+^ adsorbs, as follows from eq 47. FF MD simulations with optimized force fields quantitatively reproduce this trend,^77^ see the symbols in Figure 15(A).

Other possible explanations for the negative surface charge on the vapor/water interface include the adsorption of charged surfactants, which adsorb in significant quantities even at minute bulk concentrations. For example, even nanomolar concentrations of SDS and C_12_TAC strongly affect sum-frequency spectra of the CCl_4_/water interface.^186,187^ Adsorption of charged species that are present only at low concentrations can be conveniently detected by studying the change of surface tension at low salt concentration. It has been known for a long time that the surface tension γ of the vapor/water interface exhibits a minimum at a concentration of about 1 mM of salt, largely independent of salt type, known as the Jones–Ray effect.^180,188^ In Figure 15(B), we show the experimental results for the surface tension change Δγ as a function of the NaCl concentration from different laboratories using different methods. To explain the minimum, the surface tension at the vapor/water interface including H_3_O^+^, OH^–^ and surface-active charged impurities is calculated by solving the extended Poisson–Boltzmann equations 27–31 with a constant dielectric response ε_⊥_(*z*) = ε, no steric interactions and using the ion-surface interaction μ_i_^*^(*z*) from eq 23.^189^ The surface tension is calculated by integrating eq 45,
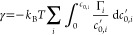
48where the logarithmic derivative of the activity in eq 45 vanishes because the activity tends to the concentration at low concentration. To parametrize μ_i_^*^(*z*), the Gibbs dividing surface is used for *z*^⧧^, z* = 0.5 nm, and the values of ν_*i*_ for Na^+^, Cl^–^, H_3_O^+^ and OH^–^ are determined by fitting eq 48 to the experimental surface tension as a function of the concentration. The results of the fit are ν_Na^+^_ = 1.2, ν_Cl^–^_ = 1.0 and ν_OH^–^_ = 1.6, all being repelled from the interface, and , showing slight adsorption. The bulk concentrations of H_3_O^+^ and OH^–^ are equal to  at neutral pH = 7. The result for Δγ as a function of the NaCl concentration is shown as a broken line in Figure 15(B), clearly failing to reproduce the experimental results at low salt concentration.

Because only H_3_O^+^, OH^–^, Na^+^ and Cl^–^ are insufficient to reproduce the Jones–Ray effect, a fifth species is added to model the presence of impurities. It has been shown experimentally that intentionally adding small amounts of the surfactant C_12_TAB indeed induces the Jones–Ray effect.^190,191^ The impurities are parametrized by fitting the surface tension of the common surfactant SDS, leading to an estimated value of ν_*imp*_ = −15.6, similar to the value for other surfactants.^189^ The bulk concentration of impurities *c*_0,*imp*_ is treated as a fit parameter. By adding impurities with a bulk concentration in the nanomolar range, the experimental data can be fitted with very high accuracy, see Figure 15(B). Note that different impurity concentrations are necessary to fit the data sets from different measurement methods and laboratories, which supports the hypothesis that impurities cause the Jones–Ray effect.

Summarizing, hydroxide adsorption does not provide a consistent explanation for the surface tension of the vapor/water interface at low and high salt concentrations. In fact, hydroxide does not adsorb to the interface at all. Also the presence of hydronium ions, although they do adsorb to the interface, does not explain the Jones–Ray effect because the adsorption potential is insufficiently strong. Instead, minute quantities of charged surfactants do explain the experimental measurements using only the bulk impurity concentration as a fit parameter. Because of the strong adsorption potential of surfactants, impurities are also predicted to have a measurable effect on the disjoining pressure^192^ and on the electrophoretic mobility.^132,193^ Trace amounts of impurities are indeed found to modify the stability of colloidal emulsions and the electrophoretic mobility of colloids.^194^ Therefore, the possible presence of impurities at concentration levels that are almost impossible to detect should be taken into account when modeling experimental results for hydrophobic surfaces in aqueous solution at low salt concentrations.

## 5 Discussion and Outlook

In the preceding sections we have reviewed a scheme for the development of multiscale models of the electric double layer. Starting from quantum DFT-based and FF-based MD simulations, continuum response functions and effective potentials are calculated and inserted into extended Poisson, Boltzmann and Stokes equations. This approach can be seen foremost as a practical way to substantially improve the predictive power of the Poisson–Boltzmann equation. Of course, by extending the Poisson–Boltzmann equation, the simple analytical solutions that make the Gouy–Chapman theory so alluring are typically lost. However, making some rational approximations of the simulated profiles, e.g., by choosing box profiles that can be parametrized based on simulations or experiments, analytical solutions often still exist. The primary insight offered by incorporating the molecular simulations concerns the important role played by the interfacial water structure, which is largely independent of the surface charge density and the presence of ions. In particular, simulations show that many effects of the Stern layer—often interpreted as a layer of adsorbed ions—are caused by the structure imposed on pure water by the sheer presence of the interface. For example, the steep rise of the electrostatic potential near charged interfaces, steeper than predicted by the Gouy–Chapman theory, is caused by the water structure, the effect of which is represented by the dielectric response function. Also the enhanced or reduced viscous friction in the interfacial layer at hydrophilic and hydrophobic surfaces, respectively, can be reproduced in simulations of pure water at uncharged surfaces. In contrast, adsorbed, partially dehydrated ions and the formation of an inner Helmholtz layer are only found at very high surface charge densities, with an absolute value well exceeding 1*e*/nm^2^.

In the multiscale approach discussed, the effects of the molecular structure are incorporated at the continuum level with a number of approximations. Important approximations concern the assumption of locality of the response functions and the incorporation of effective ion–surface interaction potentials directly in the Boltzmann equation. Furthermore, fluctuations and correlations between particles are not included, and neither is the influence of the nonideality of the electrolyte activity accounted for in most treatments. Electronic effects originating in the substrate, which are known to affect double layer electrostatics at metal interfaces,^195,196^ are not considered in the FF MD simulations. In particular, apart from the variable position of the solvent molecules with respect to the metal, the capacitance of metal electrodes as a function of charge is expected to depend sensitively on the center of mass of the excess charge distribution and the dependence of the interfacial dielectric response on the electrode charge.^195^ Surprisingly, however, classical methods to simulate metallic interfaces^197^ show that ion–surface interactions and local dielectric properties at metallic graphite/water interfaces are almost identical to the results at nonmetallic graphite.^119^ These potentiostatic classical MD simulations model the redistribution of charge in the electrode, but the charge is still located on the metal atoms, whereas the spatial extent of the charge response is determined by the electron density, typically extending beyond the atom positions. One way to better account for polarization effects at the interface in MD simulations is treating the solid using electronic structure methods,^198^ which additionally allows for electrochemical reaction modeling.^199^ For computational efficiency, the electrolyte can be treated on a more coarse-grained level, for example, using force fields, equilibrium distributions from classical density functional theory or continuum models.^200^ In the latter case, different combinations of nonlinear, nonlocal, and anisotropic models for the dielectric response are available to model the polarizable medium constituting the continuum.^200^ The effect of metallicity on interfacial viscosity is so far unexplored, but recent results suggest an effect of metallicity on surface friction.^201^ Although atomic-scale inhomogeneity is included in the simulations, the surfaces are treated as laterally homogeneous in the continuum theory, which may be insufficient in some cases.^154,202^ Finally, a topic that is of crucial importance for the further development of the multiscale framework is the availability of accurate force fields for the MD simulations. Although methods to develop ion force fields in bulk water exist and perform well at the vapor/water interface,^48,77^ these force fields have not been tested extensively on different types of surfaces. An added complication is that whereas the optimization targets for the ion–water and ion–ion interactions are clear, experimental reference quantities for the parametrization of force fields for the substrates are less readily available. If it turns out to be necessary, alternative force field parametrizations in regions of varying dielectric properties and near interfaces are being developed.^203,204^ In the past decade, new simulation methods based on machine-learned (ML) potentials developed rapidly because they reach DFT accuracy at a fraction of the computational costs.^205−208^ Originally, ML potentials were purely short-range, but recent extensions have made them also able to cover long-range effects, such as electrostatics,^208−210^ which are crucial for simulating the electric double layer. First studies already show that ML potentials mitigate some of the force field accuracy problems and DFT MD time-length scale issues of aqueous systems.^211,212^ Similarly to force field parametrizations, however, the choice of exchange and correlation functionals when using DFT MD simulations is essential for the success of the approach reviewed here.^213,214^

Given these limitations, assumptions and approximations, the results of the simulations and continuum calculations are compared to experimental results for the interfacial molecular orientation, interface potential, double layer capacitance, electrokinetic flow, interfacial viscosity, surface tension and disjoining pressure. Specific conclusions from the comparison with experiments can be summarized as follows.

FF- and DFT-based simulations and sum-frequency generation experiments of the vapor/water interface give a similar picture of the interfacial water structure regarding the molecular density and orientation. DFT-based simulations provide an estimate of the mean inner potential of bulk water, which agrees with the most recent experimental results. Both FF- and DFT-based simulations show that the electrochemical potential difference across an interface is significantly smaller than the unperturbed water potential found in simulations, but an accurate numerical value is still elusive.

The dielectric and viscous properties of the interfacial layer can be calculated from FF-based MD simulations using simple rigid water models. The anisotropic dielectric environment at the interface can be used to calculate the linear part (depending quadratically on the ionic charge) of the electrostatic energy of ions at dielectric boundaries, but the surface-specific nonlinear contributions are important in the interfacial region. The extended Poisson–Boltzmann equation, including the dielectric profile and using a heuristic nonelectrostatic potential, succeeds at reproducing simulated ion density profiles at solid and lipid interfaces, with and without surface charge. The same framework accurately reproduces experimental results for the interfacial capacitance as a function of salt concentration and as a function of confinement in a slit geometry, using the effective slit width as a fit parameter.

Although the viscosity at hydrophilic and charged hydrophobic surfaces is strongly enhanced, a truly stagnant layer only forms at surface charge densities well exceeding 1*e*/nm^2^ in absolute value for nonpolar surfaces. In that case, the stagnant layer coincides with the inner Helmholtz layer. Experimental results for the electrophoretic mobility of colloids can be accurately reproduced by combining the Stokes and Poisson–Boltzmann equations with the simulated dielectric and viscous response profiles.

Comparing the extended Poisson–Boltzmann equation to electrokinetic experiments, we estimate based on the bulk electrolyte properties that the presence of ions, as well as the strong electric field in the interfacial layer at charged surfaces, have only a minor influence on the interfacial dielectric and viscous properties. Nevertheless, adsorbing ions and nonzero surface charges can have strong effects on the orientation of water molecules in the interfacial region.^74^ Adsorbing ionic species can also give rise to a significant surface charge density. In particular, the presence of minute quantities of ionic surfactants are found to have a measurable effect on the surface tension, the disjoining pressure between surfaces and the electrophoretic mobility of air bubbles.

In conclusion, combining molecular simulations and continuum theory enables us to make reliable predictions without computationally prohibitive calculations. Apart from these practical considerations, the different modifications in the extended Poisson–Boltzmann equation do allow us to unravel the different components of the theory underlying the various experimental observations, as the summary above clearly shows.
